# The Role of Lipid Metabolism in Gastric Cancer

**DOI:** 10.3389/fonc.2022.916661

**Published:** 2022-06-15

**Authors:** Meng-Ying Cui, Xing Yi, Dan-Xia Zhu, Jun Wu

**Affiliations:** Department of Oncology, The Third Affiliated Hospital of Soochow University, Changzhou, China

**Keywords:** gastric cancer, lipid metabolism, biomarkers, treatment, chemoresistance

## Abstract

Gastric cancer has been one of the most common cancers worldwide with extensive metastasis and high mortality. Chemotherapy has been found as a main treatment for metastatic gastric cancer, whereas drug resistance limits the effectiveness of chemotherapy and leads to treatment failure. Chemotherapy resistance in gastric cancer has a complex and multifactorial mechanism, among which lipid metabolism plays a vital role. Increased synthesis of new lipids or uptake of exogenous lipids can facilitate the rapid growth of cancer cells and tumor formation. Lipids form the structural basis of biofilms while serving as signal molecules and energy sources. It is noteworthy that lipid metabolism is capable of inducing drug resistance in gastric cancer cells by reshaping the tumor micro-environment. In this study, new mechanisms of lipid metabolism in gastric cancer and the metabolic pathways correlated with chemotherapy resistance are reviewed. In particular, we discuss the effects of lipid metabolism on autophagy, biomarkers treatment and drug resistance in gastric cancer from the perspective of lipid metabolism. In brief, new insights can be gained into the development of promising therapies through an in-depth investigation of the mechanism of lipid metabolism reprogramming and resensitization to chemotherapy in gastric cancer cells, and scientific treatment can be provided by applying lipid-key enzyme inhibitors as cancer chemical sensitizers in clinical settings.

## Background

Despite significant advances in clinical practice over the past few years, gastric cancer (GC) remains recognized as the leading cause of cancer-related deaths worldwide. Chemotherapy takes on a critical significance in the treatment of local and metastatic gastric cancer, whereas chemotherapy resistance has limited the efficacy of chemotherapy. Chemotherapeutic drug resistance was found as a complex multi-factor phenomenon, whether inherent or acquired, which was linked to tumor cells and tumor microenvironment (1). Abnormal lipid metabolism has been found as one of the critical metabolic features of tumor cells. In addition, cancer cells obtain fatty acid through lipid decomposition, thereby promoting fatty acid (FA) synthesis and increasing lipid uptake. FAs were found to play a certain role in various aspects of tumorigenesis and tumor progression (2). However, the specific process of lipid reprogramming in gastric cancer remains unclear, and the biological functions and regulatory mechanisms of lipid reprogramming have been rarely investigated. Under normal physiological conditions, gastric epithelial cells are constantly renewed. When damages to the stomach due to various factors occur, the epithelial cells tend to renew more rapidly. During the occurrence of gastric cancer, gastric epithelial cells proliferated rapidly, leading to the transformation from normal gastric mucosa to atrophic gastritis, intestinal metaplasia, dysplasia, as well as gastric cancer (3). Abnormal lipid metabolism can affect all aspects of gastric cancer growth. An abnormal expression of genes correlated with fatty acid synthesis or oxidation was found to be significantly correlated with malignant phenotypes (e.g., tumor metastasis, drug resistance, and recurrence) (4). Low serum high-density lipoprotein levels predicted a higher risk of gastric cancer, higher rates of lymphatic and vascular infiltration, advanced lymph node metastasis, as well as poor prognosis (5–7). Adipocytes and fatty acids were found to promote gastric cancer metastasis, which indicated a poor prognosis of gastric cancer (8–10). In a mouse gastric cancer model, it was found that tumor tissues were often characterized by the up-regulated expression of fatty acid metabolism-related proteins (11, 12). Accordingly, targeting lipid metabolism has been recognized as a promising strategy for the treatment of gastric cancer patients. However, specific strategies targeting the vital regulatory factors for lipid metabolism reprogramming to treat gastric cancer remain challenging in existing medical research. In this study, the mechanisms of lipid metabolism in gastric cancer cells are briefly reviewed, with an emphasis on the pathways of lipid origin, utilization and storage, as well as the vital enzymes of lipid metabolism used as biomarkers for monitoring gastric cancer metastasis, therapeutic effect and prognosis. Targeting and blocking pathways in gastric cancer can enhance chemotherapeutic resistance and increase possible therapeutic benefits. This study aims to identify novel prognostic biomarkers to reduce risk, stratify patients, and guide subsequent research into potential new therapeutic targets.

## Reprogramming of Lipid Metabolism in Gastric Cancer

### Exogenous FAs Absorption

CD36 accounts for free fatty acid and cholesterol uptake and intracellular signaling, playing a critical role in cancer-related antigen presentation, inflammation and angiogenesis (13). CD36 was found to increase the uptake of exogenous palmitic acid in gastric cancer and induce metastasis *via* the AKT/GSK-3β/β-catenin signaling pathway (14). Furthermore, fatty acid-induced up-regulation of CD36 led to an increase in the fat uptake by GC cells, thus leading to a formation of a vicious cycle that could promote GC metastasis (10). The results of this study suggest that CD36 may serve as a potential therapeutic target for metastatic gastric cancer. Absorption of fatty acids is also aided by fatty acid-binding proteins (FABPs), a family of proteins involved in the regulation of FAs storage and distribution (15). FABP5, members of the FABPs family, was correlated with tumorigenesis. Zhao et al. found that FABP-5 was involved in gastric cancer cell cycle regulation and apoptosis, and its expression level was significantly correlated with the invasiveness of gastric cancer cells (16).

### *De Novo* Synthesis of Lipids

Acetyl-CoA is produced by ATP citrate lyase (ACLY) catalyzing the conversion of citric acid into oxaloacetic acid in the cytoplasm, which has been found as a significant component of endogenous FAs and cholesterol biosynthesis. ACLY is often highly expressed in gastric adenocarcinoma patients (17). Acetyl-CoA synthase 2 (ACSS2) is an enzyme catalyzing the conversion of acetate to acetyl-CoA. It is overexpressed under hypoxia and lipid reduction conditions. ACSS2 has been found as the only enzyme that recycles acetate from cytoplasmic and nuclear deacetylation (e.g., deacetylation of proteins and metabolites) (18). Surprisingly, loss of ACSS2 expression was identified in a high proportion of gastric cancers (62.6%), whereas this was more commonly observed in poorly differentiated gastric adenocarcinoma or signet ring cell carcinoma (19). Cancer cells form a capable nascent FAS mechanism with the increased activity of key adipogenic enzymes (e.g., acetyl-CoA carboxylase (ACC) and fatty acid synthase (FASN)). He et al. reported a significant negative correlation between ACC expression and the immune characteristics in GC, which revealed that the inhibition of ACC could enhance anti-tumor immunity in GC (20). Besides, as revealed by the result of the experimental study conducted by Fang et al., with the advancement of disease stage and lymph node metastasis, the expression of the phosphorylated form of ACC decreased, which further supported the key role of ACC in the occurrence and development of GC (21). *De novo* lipogenesis can produce saturated fatty acids, which are converted to polyunsaturated fatty acids (MUFAs) *via* stearoyl-CoA desaturases (SCDs), the preferred substrates for triacylglycerols (TAGs) formation. Tumor cell proliferation is significantly dependent on MUFAs; in the absence of exogenous sources of MUFAs, they are completely dependent on SCD1 activity (22). By conducting gene expression manipulation and bioinformatics analysis, Wang et al. suggested that SCD1 could facilitate the migration of gastric cancer cells and anti-iron cell apoptosis and growth, illustrating the potential of SCD1 as a therapeutic target for gastric cancer (23). However, based on the needs of tumor cells, synthetic monounsaturated fatty acids are preferentially converted to triglycerides for adipose tissue energy storage, or phospholipids for membrane formation and signaling functions (24). The expression levels of the aforementioned enzymes are regulated by sterol regulatory element binding protein 1 (SREBP-1), which is a major transcription factor that controls lipid metabolism. In GC, activation of sterol regulatory element-binding protein 1 c (SREBP-1c) leads to changes in lipogenic enzymes, including SCD1 and FASN up-regulation and fatty acid elongase 6 (ELOVL6) down-regulation. The combined action of these enzymes was found to lead to a reduction in phosphatidic acid (PA), which was treated in gastric cancer cells at high concentrations (25).

### Formation and lipolysis of lipid droplets

Cancer cells are characterized by the up-regulation of fatty acid synthesis (FAS) and FAs uptake, which lead to an increased LDs accumulation, and in gastric cancer, this is not an exception. As reported by Enjoji et al., fat production and lipid storage generally increased in the process of gastric carcinogenesis, and SREBP1c and DGAT2 genes were also significantly up-regulated. In contrast, genes promoting intracellular lipid consumption/removal (β-oxidation and lipolysis) were consistently down-regulated in tumor mucosa (26). Excessive fatty acids and cholesterol in cells can be converted to triglycerides (TGs) and cholesteryl esters (CEs) by diacylglycerol O-acyltransferase 1/2 (DGAT1/2) and sterol O-acyltransferase 1 (SOAT1)/Acetyl-CoA acyltransferase 1 (ACAT1) to form LDs. He et al. suggested that a high expression of DGAT1 led to lower overall survival in poorly differentiated gastric cancer patients. Inhibition of DGAT1 may serve as a promising strategy for gastric cancer therapy (27). As revealed by another experimental study, adipocytes could provide FAs to GC cells, thereby facilitating NADPH synthesis and anoikis resistance. The above adipocyte-derived FAs are transported to GC cells to form lipid droplets, followed by the induction of re-esterification of adipocyte FAs by DGAT2. During peritoneal metastasis, the up-regulation of DGAT2 increased intracellular lipid metabolism and provided NADPH for scavenging reactive oxygen species (ROS) (28).

It was found that excessive lipids in cells did not exist as non-esterified free fatty acids (FFAs) since high concentrations of FFAs are potentially cytotoxic (29). As a result, cells stored excessive fatty acids and cholesterol in neutral, inert biomolecules (e.g., sterols and TG) in cellular structures, termed lipid droplets or LDs (30, 31). LDs were deposited inside the cell by lipids surrounded by a layer of phospholipids; lastly, structural proteins termed perilipins (PLINs) were separated from the hydrophilic cytoplasm (32–34). Perilipin2 (PLIN2), also known as adipose differentiation-related protein (ADRP), is a member of the tail-interacting protein (PAT) family of PLIN, which involved in lipid droplet formation (35). Sun et al. confirmed that periilipin2 could facilitate the regulation of gastric cancer cell proliferation and apoptosis by inhibiting ferroptosis (36).

Lipid droplets are dynamic organelles that can also be hydrolyzed by intracellular lipase to release FAs when required (e.g., under nutritional stress). Adipose triglyceride lipase (ATGL) initiated degradation of intracellular TAG and hydrolyze TAG to generate diacylglycerol (DAG). Al-zoughbi et al. found that low ATGL mRNA levels were correlated with significantly reduced survival in patients with gastric cancer (37). DAG is hydrolyzed by hormone-sensitive lipase (HSL) to generate monoacylglycerol (MAG). Monoacylglycerol lipase (MAGL) was found to hydrolyze MAG, release the glycerol backbone, and release FAs (38). In gastrointestinal stromal tumors (GISTs), overexpression of monoglyceride lipase (MGLL) is correlated with adverse clinicopathological factors, suggesting a pathogenic role in the aggressive phenotype of primary localized gastrointestinal stromal tumors. The study by Li et al. confirmed the disturbance of cellular lipid metabolism in gastrointestinal stromal tumors and identified MGLL as the top candidate gene playing a crucial role in the progression of GIST (39). Besides, lipolysis stimulated lipoprotein receptor (LSR) was found as a lipoprotein receptor binding to triglyceride-rich lipoproteins with increased affinity when activated by FFAs (40). It was found that inhibition of LSR reduced lipid droplet storage, which revealed that a high expression of LSR could up-regulate lipid metabolism (41). Consistently, Sugase et al. reported that the Janus kinase/signal transduction and transcription activator (JAK/STAT) and phosphatidylinositol-3-kinase (PI3K) signaling pathways were enhanced after administration of low-density lipoprotein (VLDL) but inhibited in GC cells. LSR was inhibited by monoclonal antibody (#1-25) against human LSR (42). Lipid metabolism plays a role in GC cell proliferation *via* LSR. Notably, lipid droplets can serve as a tumor-promoting source of FAs. FA lipolysis mediated by MAGL has been reported to facilitate tumor migration, invasion, survival, and growth. Therefore, targeting FAs esterification and lipolysis are considered potential therapeutic strategies requiring further investigation.

### Cholesterol Synthesis

In mammalian cells, cholesterol is synthesized from acetyl-CoA *via* the mevalonate acid pathway. First, 3-hydroxy-3-methylglutaryl coenzyme A (HMG-CoA) is synthesized from three acetyl-CoA molecules. HMG-CoA is reduced to mevalonate acid under the action of HMG-CoA reductase (HMGCR). As reported by Li et al., HMGCR activates Hedgehog/Gli1 gene expression that promotes Gli1, thereby facilitating the growth and migration of gastric cancer cells (43). In an enzymatic reaction, mevalonate acid is converted to Farnesyl diphosphate (FPP). Two FPP molecules condense to squalene. Subsequently, squalene is oxidized by squalene epoxidase (SQLE) to 2,3-oxidosqualene, which is cycled to lanosterol. Lanosterol is ultimately converted to cholesterol (44). Primary and metastatic gastric cancer cells exhibit different sensitivities to drugs affecting isoprenoid synthesis, metabolism, and cholesterol uptake. Isoprenoids play a vital role in the growth and survival of the above two types of cells, whereas the effects of free cholesterol and esterified cholesterol on the survival of metastatic gastric cancer cells are not as significant as those of primary gastric cancer cells. Differences in low-density lipoprotein receptor (LDLR) expression due to mevalonate acid pathway inhibition suggest different cholesterol uptake regulation between primary and metastatic cancer cells (45). Besides cholesterol biosynthesis, most cells acquire extracellular cholesterol through LDLR (46). LDLR refers to a transmembrane protein that mediates cellular cholesterol uptake. Plasma oxidized low-density lipoprotein (oxLDL) is considered a risk factor for tumor development in patients with abnormal lipid metabolism. LDL binds to LDL, transporting cholesterol to cancer cells. Ox-LDL up-regulates the expression of vascular endothelial growth factor C (VEGF-C) by binding to lectin-like oxidized low -density lipoprotein receptor-1 (LOX-1) -mediated nuclear factor κB (NF-κB) signaling pathway, it also interacts with a cluster of differentiated cells (CD36). Lastly, proliferation and distant metastasis of gastric cancer cells were induced (47).

Excessive cholesterol is exported from cells by ATP-binding box transporters, including ATP-binding box transporter A1 (ABCA1) and ATP-binding box G transporters (ABCGs), or by sterol O-acyltransferase 1/acyl-CoA: cholesterol acyltransferase (SOAT1/ACATs) transformed into more harmful cholesterol (48). As reported by Zhu et al., SOAT1 up-regulated cholesterol metabolism gene sterol regulatory element-binding protein 1 (SREBP1) in the development of gastric cancer. SREBP1 and sterol regulatory element-binding protein 2 (SREBP2) induce lymphatic angiogenesis by increasing VEGF-C expression. Overexpression of SOAT1 enhanced the proliferation, migration, and invasion of GC cells (49). The above CEs are stored in LDs or secreted into lipoproteins. Chang et al. found that lipoprotein-mediated cholesterol entry and steroid production are biological signals that promote gastric cancer progression (50).

Cholesterol concentrations are regulated by SREBP-2. The oxysterol receptor liver X receptor α and β (LXRα and LXRβ) are nuclear receptors having a significant effect on the transcriptional control of lipid metabolism. Transcriptional activity of Liver X receptors (LXRs) is induced in response to the increase in cellular cholesterol levels. LXRs are capable of binding to and regulating the expression of genes encoding cholesterol absorption, transportation, outflow, excretion, and conversion to bile acids. Furthermore, LXRs were found to regulate fatty acid metabolism by adjusting the adipose formation transcription factor sterol regulatory element-binding protein 1c and genes encoding fatty acid elongation and desaturation proteins (51). LDLR expression is regulated at the transcriptional and post-translational levels. SREBP2 serves as a major transcription factor for LDLR. In response to low cholesterol levels, SREBP2 drove the transcription of genes encoding proteins participating in cholesterol biosynthesis and the uptake of LDL cholesterol (52). LXRβ agonists inhibit GC cell proliferation by inhibiting Wnt signaling through LXRβ relocalization (53). Treatment with activated LXRβ and paclitaxel could inhibit the proliferation and induce the apoptosis of gastric cancer cells and up-regulate the expression of activated transcription factor 4 (ATF4) in a concentration-dependent manner (54).

### Synthesis of Long-Chain Acyl-CoA and Fatty Acids Oxidation (FAO)

The Acyl-CoA synthetase long-chain family members (ACSL) play a part in essential lipid metabolism pathways (e.g., the peroxisome adipocytokine signaling pathway and fatty acid metabolism). It is therefore revealed that the ACSL family is a vital determinant of lipid metabolism. Acyl-CoA synthetase long-chain family member (ACSL4) is one of the isoforms of long-chain fatty acid acyl-CoA synthase. Ye et al. proved that the increase or decrease of ACSL4 expression in GC cell lines significantly affected cell proliferation and migration, which revealed that ACSL4 could play a vital role in inhibiting GC growth and metastasis (55). Furthermore, arachidonate 15-lipoxygenase (ALOX15) refers to a polyunsaturated fatty acid metabolism enzyme that, similar to other lipoxygenases, has various physiological and pathological central products. Kalamkari et al. reported that the ALOX15 gene product played a role in anti-inflammation, membrane remodeling, as well as cancer development and metastasis. Thyroid peptide receptors (FPR1, 2 and 3) are the pattern recognition receptor (PRR) family of G-protein-coupled receptors (GPCR), which are capable of recognizing exogenous and endogenous “danger” signals and triggering inflammation and immune response (56). Chronic inflammation can result from inadequate engagement of resolution mechanisms. This phenomenon is primarily due to the metabolic activity of lipoxygenase (ALOX5/15) on ω-6 or ω-3 essential polyunsaturated fatty acids (PUFAs) (57). Prevete et al. reported that genetic modulation of formylpeptide receptor-1 (FPR1) in gastric cancer cells can regulate ALOX5/15 expression and increase the potential for angiogenesis and tumorigenesis (58). Liu et al. suggested that Honokiol inhibited gastric carcinogenesis by activating 15-lipoxygenase-1 (15-LOX-1) and subsequently inhibiting peroxisome proliferator-activated receptor-g (PPAR-g) and cox-2-dependent signal transduction. 15-LOX-1-regulated PPAR-g and targeting the cyclooxygenase-2 (COX-2) signaling pathways may serve as a promising therapeutic approach (59).

Fatty acids resulting from adipose decomposition are decomposed *via* the mitochondrial fatty acid β -oxidation pathway to comply with the energy requirements of rapidly proliferating cells. FAO involves a series of cyclic reactions leading to the oxidation of fatty acid beta-carbon. The respective cycle of FAO was found to result in two carbon-shortened fatty acids and the production of reduced nicotinamide adenine dinucleotide (NADH), reduced flavin adenine dinucleotide 2 (FADH2) and acetyl-CoA. NADH and FADH2 produced by FAO entered the electron transport chain (ETC) to produce ATP (60). FAO plays a vital role in satisfying the energy needs of cancer cells, which can ensure their survival and proliferation in the acidic and low-oxygen tumor environment. Ezzeddini et al. found that the up-regulation of Hypoxia-inducible factor-1α(HIF-1α) and the down-regulation of lipid catabolic genes may be parallel to the down-regulation of FAO mediated by PPAR γ in patients with gastric adenocarcinoma (GA) and primary gastric cancer. This metabolic adaptation to hypoxic conditions may contribute to the pathogenesis of GA and be of clinical and therapeutic significance in patients with GA (61).

In gastric cancer, the rate of lipogenesis increases significantly, and the level of mitochondrial fatty acid β-oxidation increases, which is largely involved in lipid rafts and lipid-modified signaling molecules, thus promoting a wide variety of signal transduction in cancer cells. Fatty acids are employed to meet the needs of cell membrane synthesis, and the degradation of fatty acids is promoted by β -oxidation, leading to the accumulation of fatty acids. Fatty acids (e.g., hexadecenoic acid, docosahexaenoic acid, valeric acid, and β-hydroxybutyric acid) are significantly more prevalent in gastric cancer than in benign tissues (e.g., chronic superficial gastritis) (62). Elevated levels of octadecanoic acid have also been detected in blood samples from patients with gastric cancer. To be specific, β-hydroxybutyric acid was found as a typical product of fatty acid degradation by β-oxidation, which indicated that fatty acid decomposition could be more intense in the microenvironment (63). As revealed by Currie’s study, fatty acid degradation pathways are activated during the development of gastric cancer. Activation of fatty acid degradation was reported as a common feature of all cancers, as cell proliferation requires fatty acids to synthesize cell membranes and label molecules (64).

Carnitine acyltransferase 1 (CPT1) is a mitochondrial outer membrane enzyme catalyzing the rate-limiting step of FAO by transporting FAs across the mitochondrial membrane. Its activity is dependent on tissue-specific needs in FA metabolism and energy consumption. Tumor cells could acquire FAs through lipolysis, thus facilitating tumor proliferation, survival, drug resistance, as well as stem cell formation (65–67). The experimental study of Wang et al. revealed that overexpression of carnitine acyltransferase 1 A (CPT1A) was correlated with pathological stage, lymph node metastasis and poor prognosis in gastric cancer patients. Overexpression of CPT1A also facilitated the proliferation, invasion and EMT processes of GC cells (68). Chen et al. found that hypoxia-induced high expression of carnitine acyltransferase 1 C(CPT1C) is closely correlated with poor prognosis and can promote GC cell proliferation (69).

**Figure 1:**
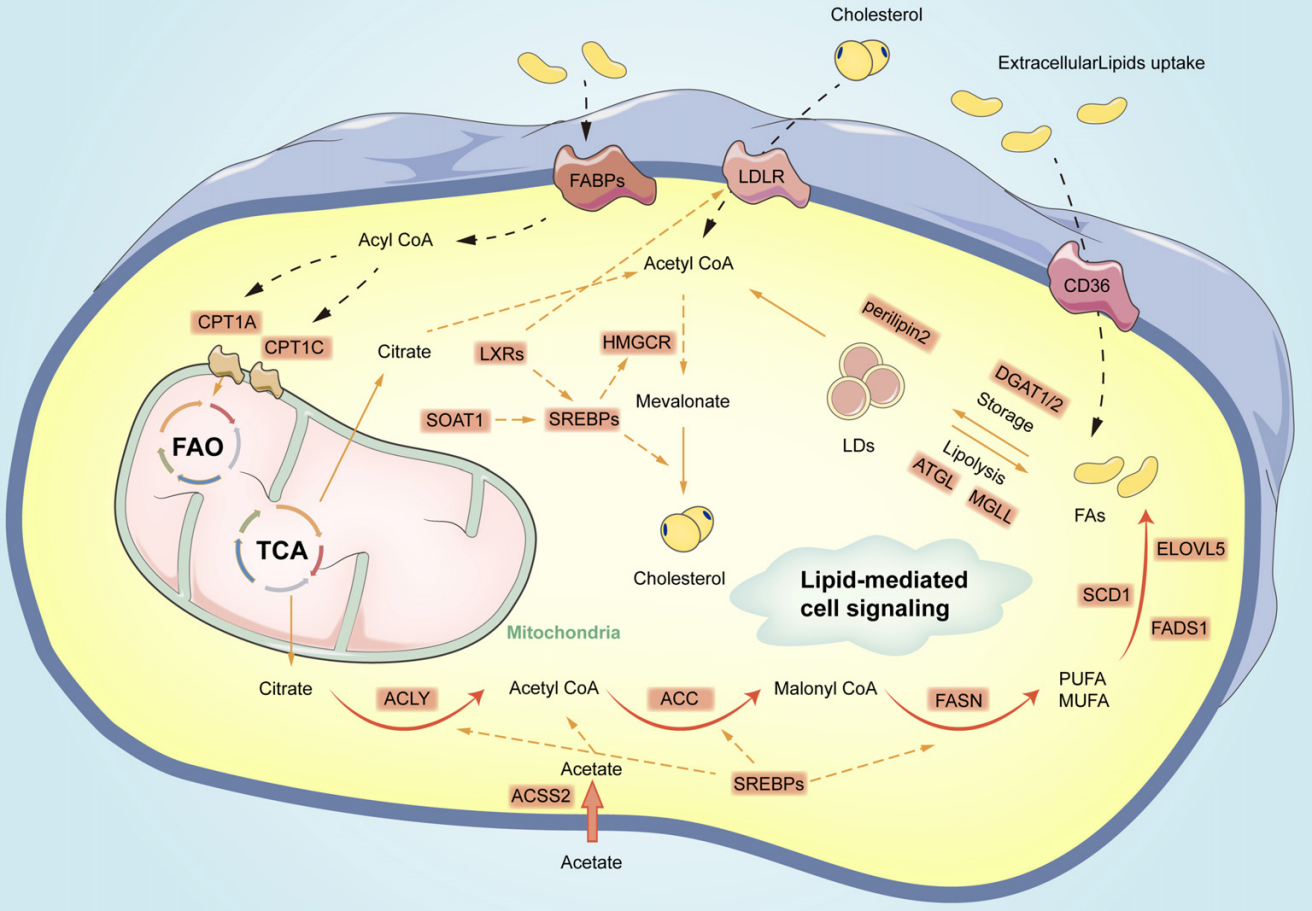
Overview of fatty acid metabolism reprogramming in gastric cancer cells. The process primarily includes *de novo* lipid synthesis, fatty acid oxidation, cholesterol synthesis, formation, as well as lipolysis of lipid droplets. Orange circles represent vital enzymes in lipid metabolism pathways. CPT1, carnitine palmitoyl transferase 1; FAs, fatty acids; FAO, fatty acid oxidation; TCA, Tricarboxylic Acid; LDs, lipid droplets; MUFA, Mono-unsaturated fatty acids; PUFA, polyunsaturated fatty acids; DGAT1/2, diacylglycerol O-acyltransferases 2; ATGL, Adipose triglyceride lipase; MGLL, monoglyceride lipase; ELOVL5, elongation of very long-chain fatty acid protein 5; FADS1, fatty acid desaturase 1; SOAT1, Sterol O-acyltransferase 1; LXRs, Liver X receptors; CD36, Cluster of differentiation 36; LDLR, low-density lipoprotein receptor; FABPs, fatty acid-binding proteins; ACSS2, acetyl-CoA synthetase 2;CPT1A, carnitine acyltransferase 1 A; CPT1C, carnitine acyltransferase 1 C; ATGL, adipose triglyceride lipase; HSL, hormone-sensitive lipase; MGLL, monoglyceride lipase.

Furthermore, acyl-CoA thioesterase (ACOT) is involved in FAO. ACOT is capable of catalyzing the hydrolysis of acyl-CoA to the corresponding non-esterified fatty acids and coenzyme A (CoA-SH). The above enzymes play their role by maintaining cellular levels and appropriate proportions of free and activated fatty acids and CoA-SH. Wang et al. recently found that ACOT1 was abnormally overexpressed in gastric cancer tissues, which significantly correlated with a poor prognosis of gastric cancer patients (70). Li et al. reported that the overexpression of ACOT4 in cancer-associated fibroblasts (CAFs) was correlated with the survival rate of GC patients. Individualized therapy targeting ACOT4 might also be a promising modality for the treatment of gastric cancer patients (71). (The above process is shown in **Figure 1**).

## Different Gastric Cancer Types Are Heterogenous in Terms of Lipid Metabolism

Gastric cancers are heterogeneous, and their lipid metabolism is not identical. Gastric mucosal tumors fall into two distinct main entities, the diffuse and intestinal subtypes, in accordance with Lauren’s histological criteria. Ortiz et al. suggested that LDL receptor levels were lower in primary gastric tumor tissue, but only in the diffuse type (72). As revealed by Karthik et al., the intestinal subtype of gastric cancer was primarily dependent on glucose and glutamine metabolism, and the diffuse subtype of gastric cancer relied heavily on dysregulated fatty acid metabolism. Most fatty acid metabolism genes were enriched in diffuse subtype gastric tumors. Notably, fatty acid biosynthesis, fatty acid oxidation, fatty acid transport, and fat differentiation (fatty acid storage) genes were enriched in the diffuse subtype of gastric tumors. Gastric tumors of the diffuse subtype also achieve a high expression of fatty acid metabolism genes (e.g., FASN, ACC, and ACLY). Two distinct metabolic alterations have been identified to play different roles in the intestinal and diffuse subtypes of gastric tumors. Moreover, possible metabolic differences between the intestinal and diffuse subtypes of epithelial and mesenchymal gastric cancers have been proposed (73). The regulation of cholesterol uptake also differs between primary and metastatic cancer cells. Several advanced and metastatic cancer cells may tend to synthesize rather than absorb new cholesterol to provide supply. Primary and metastatic gastric cancer cells have shown different sensitivities to drugs affecting isoprenoid synthesis and cholesterol metabolic uptake. This phenomenon suggests that isoprenoids contribute to the growth and viability of the above two types of cells. However, the effects of free cholesterol and esterified cholesterol on the survival of metastatic gastric cancer cells are not as significant as those on primary gastric cancer cells (45). Zhu et al. identified four specific gastric cancer subgroups, including cholesterol-derived, glycolytic, mixed, and resting, based on cholesterol production and glycolysis pathways. The cholesterol subtypes have been found to have the worst prognosis in gastric cancer. The abnormal expression of TP53 and MYC may facilitate the malignant progression of gastric cancer by facilitating cholesterol synthesis and affecting the use of cholesterol. From the genotyping of the metabolic genes of gastric cancer, differences may exist in patients subjected to different metabolic subtypes of gastric cancer in clinical characteristics (e.g., survival), and a clinically feasible gastric cancer grading scheme can be developed to guide the design of gastric cancer targeted therapy (74).

## The Signaling Pathways Correlated With Lipid Metabolism in Gastric Cancer

### PI3K/Akt/mTOR Signal Pathway

It has been widely recognized that the accumulation of genetic alterations leads to the development of gastric cancer (75). Most gene mutations in gastric cancer were correlated with changes in biological signals (e.g., the mammalian target of PI3K/Akt/mTOR pathway) (76). The PI3K signaling pathway was reported as a vital regulator of numerous critical cellular processes (e.g., cell growth, metabolism, survival, metastasis, and chemotherapy resistance) (77). SREBP1 preferentially regulates the genes related to fatty acid biosynthesis, while SREBP2 mainly regulates cholesterol pathway genes. Both fatty acid synthesis and uptake are stimulated by mTOR signals. Thus, both mTORC1 and mTORC2 stimulate the expression and proteolysis of SREBP1, a major transcription factor that induces *de novo* lipid synthesis (78). Furthermore, mTORC1 was found to promote SREBP1 activity by preventing the nuclear entry of LIPIN1, the negative regulator of SREBP1, while mTORC2 promoted the expression of AKT, SREBP1’s downstream effector (79, 80), while preventing SREBP1 degradation in cancer cells (81). In gastric cancer, the activation of SREBP-1c could lead to changes in lipogenic enzymes (e.g., up-regulation of SCD1 and FASN) (25).

### Hippo Signal Pathway

YAP and TAZ, two key mediators of the Hippo pathway, play critical roles in carcinogenic signal transduction and cell attachment and their expression levels were significantly correlated with lipid metabolism. Abnormal regulation of the Hippo pathway was found to facilitate the proliferation and metastasis of gastric cancer, and the inhibition of the Hippo pathway effector proteins YAP and TAZ have shown important application prospects in treating gastric cancer (38). YAP/TAZ has been reported to interact with mature SREBPs in the nucleus, resulting in enhanced transcriptional activity and up-regulated expression of downstream targets (e.g., HMGCR and fatty acid synthase (FAS)) (82). In brief, the activity of mevalonate pathway-associated oncogenic proteins (YAP/TAZ) was activated by SREBP2 (83), which in turn stabilize lipid-producing enzymes and localize to the nucleus (84). Besides the typical hippo signaling pathway, the atypical Hippo signaling pathway also contributes to the reprogramming of lipid metabolism. LATS2 could inhibit the maturation of SREBPs by interacting with SREBPs precursors located in the endoplasmic reticulum to regulate lipid metabolism in a YAP/TAZ-independent manner (85).

### Wnt/β-Catenin Signal Pathway

The Wnt/β-catenin pathway has been found to play a vital role in the pathogenesis of gastric cancer, and its complex functions are significantly dependent on the transcriptional output of β-catenin (86). Activation of β-catenin correlates with the metastasis of gastric cancer. A positive feedback loop between YAP and β-catenin has been reported, and YAP/TAZ has been found to inhibit the Wnt/β-catenin signaling pathway *in vivo* and *in vitro (* 87, 88). In addition, SCD1 can facilitate the synthesis of unsaturated fatty acids. Unsaturated fatty acids activate Wnt ligands. The activated Wnt ligand could bind to FZD4 receptor, destroy the complex, stabilize the activity of β-catenin and YAP/TAZ proteins, promote the accumulation of β-catenin and YAP/TAZ proteins in the nucleus, and play a role of transcriptional regulation (89). The canonical Wnt/β-catenin pathway was found to regulate *de novo* lipogenesis (DNL) and fatty acid monounsaturation in adipocytes. Moreover, β-catenin was found to mediate the effects arising from Wnt signaling on lipid metabolism in part by the transcriptional regulation of SREBF1 (90). Simvastatin is capable of inhibiting HMGCR and down-regulating GER anylgerany L pyrophosphate (GGPP), which is a significant substrate for the post-translational modification of RhoA. Subsequently, the activity of RhoA is reduced, and reaches the inhibition level of YAP and β-catenin activity. The reduced activity level of YAP and β-catenin was found to eventually promote the apoptosis of gastric cancer cells and inhibit the proliferation and metastasis of cancer cells (91).

### Hedgehog Signal Pathway

**Figure 2:**
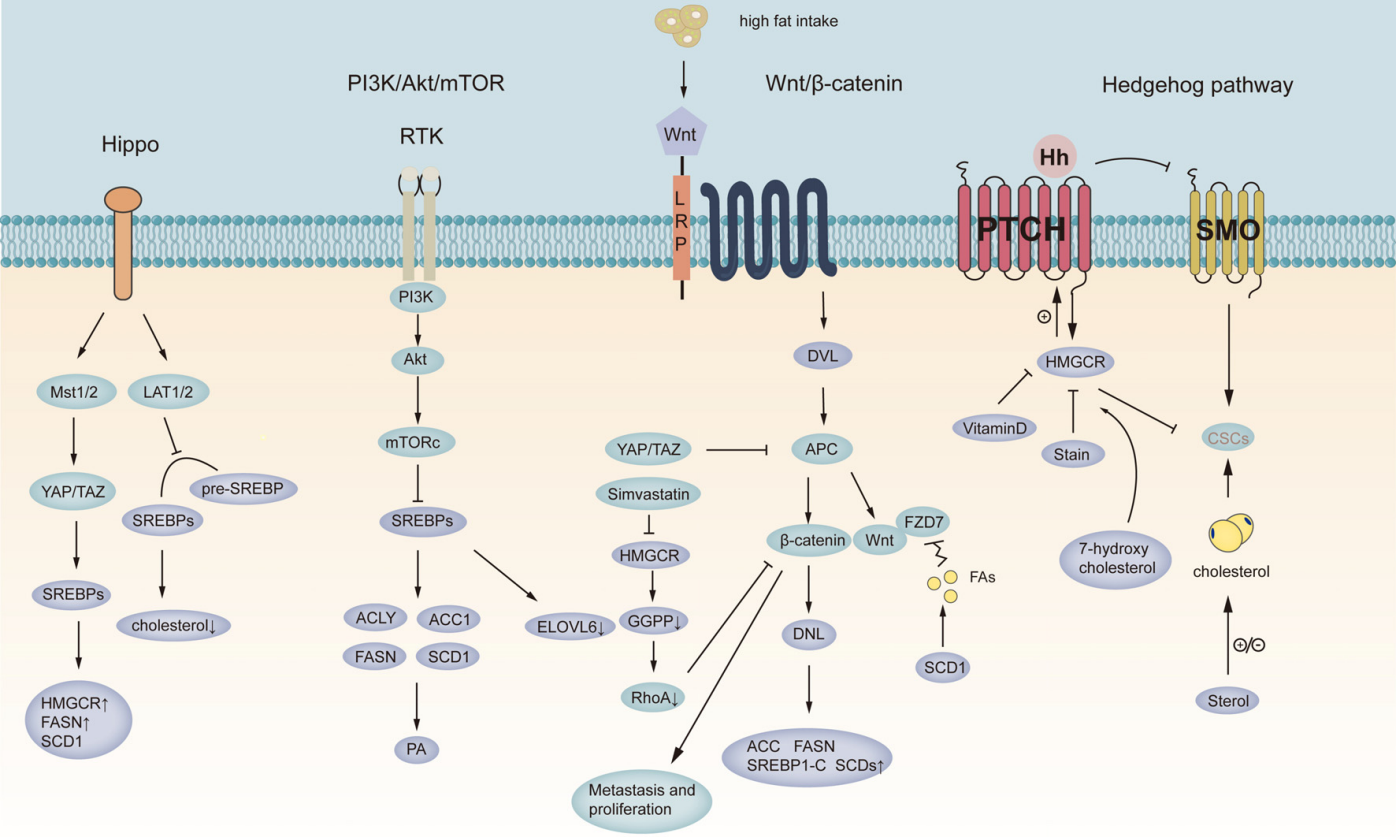
Targeting signaling pathways in gastric cancer. Schematic representation of the Wnt, hedgehog, PI-3K, and Hippo pathways in GC. The purple circles represent lipid metabolism-related pathways in gastric cancer, and the blue circles represent signaling pathways in gastric cancer. YAT/PAZ, Yes-associated protein/transcriptional coactivator with PDZ-binding motif; LAT1/2 represents Large tumor suppressor 2; MST1/2, Macrophage stimulating 1/2; SREBPs, sterol regulatory element-binding protein; Pre-SREBPs, Pre-sterol regulatory element-binding protein; HMGCR, 3-hydroxy-3-methyl-glutaryl-CoA reductase; FASN, fatty acid synthase; SCD1, stearoyl-CoA desaturase 1; ACLY, ATP-citrate lyase; ACC1, acetyl-CoA carboxylase; ELOVL6, fatty acid elongate 6; PA, phosphatidic acid; PI-3K/AKT/MTORC1, phosphoinositide 3-kinase (PI3K)-protein kinase B (AKT)-mechanistic target of rapamycin (mTOR) signaling; DVL, Doppler Velocity Log; APC, Adenomatous polyposis coli; GGPP, geranylgeranyl pyrophosphate; CSCs, cancer stem cell; FZD7, Wnt receptor frizzled7.

Sterols play a vital role in Hh protein production, secretion, transport, and Hh signal transduction. Mature Hh proteins require a double lipid modification, cholesterol necessary for Hh gradient, as well as the palmitate portion required for interactions with PTCH. When PTCH bound to Hh, the inhibition of SMO was released and activated by sterols interacting with CRD (92). The role played by cholesterol in the HH pathway has long been found since active HH proteins are covalently modified with cholesterol, which makes this post-translational modification unique among all known proteins (93). Hedgehog/Gli1 signaling pathway has been found to be frequently activated in gastric cancer. Binding hedgehog ligands to the receptor patch could activate smoothing and initiate a signaling cascade promoting the nuclear localization of Gli1 and activating the expression of downstream genomes (43). The Sonic hedgehog (SHH) pathway interacts with the genes of gastric cancer cells. Statins target HMGCR, hydroxysteroids, a rate-limiting enzyme synthesizing cholesterol, and 7-hydroxycholesterol (a precursor of vitamin D). Some metabolites of the mevalonate acid pathway have been found as active regulators of the Hh pathway, while vitamin D3 is inhibitory. Accordingly, statins were found to significantly regulate HH signaling (93). 93).(The above process is shown in **Figure 2**).

## The Function of Lipid Metabolism Related Enzymes

Disease stage and lymph node metastasis status remain the most critical determinants for designing treatment strategies and predicting prognosis in gastric cancer patients (94–97). In gastric cancer, the enzymes catalyzing the *de novo* synthesis of FAs (e.g., ACLY, ACC, FASN, and SCDs) were significantly up-regulated. The roles of the above proteins are described, and their potential as biomarkers in the diagnosis and treatment of gastric cancer is discussed.

### Lipid Fatty and Cholesterol Transporters

#### CD36

Lipid uptake from the exogenous environment has been found as another vital method for cells to acquire fatty acids besides *de novo* synthesis. CD36 was found to transport fatty acids into cells and play a vital role in cancer cell growth, metastasis, as well as epithelial-mesenchymal transition (98–100). The stomachs of CD36 mice were characterized by altered gland organization and secretion, more fibronectin, and inflammation. Other symptoms could be detected (e.g., reduced tissue respiration and mitochondrial efficiency, phospholipids increasing, as well as triglycerides decreasing). CD36 and its absence have been found to act in chronic diseases predisposing to malignancy (101).

#### FABPs

FABPs are a superfamily of lipid-binding proteins that play essential roles in fatty acid uptake, transport, as well as oxidation. They are capable of adjusting the concentration of intracellular free fatty acids by regulating the uptake and intracellular transport, maintaining a stable internal environment of lipid metabolism, and participating in the development and progression of various malignant tumors by affecting the proliferation, transformation, and migration of tumors (102–105). A high expression of FABP3 was found in the gastrointestinal stroma, which revealed that FABP3 might play a growth-promoting role in the gastrointestinal stroma. A possible mechanism for FABP3 to facilitate growth is that the proliferation of such tumors may require fatty acids formed by lipid metabolism. High expression of FABP3 was significantly correlated with poor 5-year overall survival, which indicates its prognostic value in patients with gastrointestinal stromal tumors (GISTs) (106).

The main function of FABP4 is lipid metabolism control, it plays a role in inflammatory responses, cell growth and differentiation, as well as apoptosis regulation. The expression of FABP4 is regulated by fatty acids, PPARγ, and PPARγ agonists. Lipids have essential regulatory and energy supply functions *in vivo*. Patients with elevated FABP4 expression had a poorer prognosis, and the 5-year survival rate was significantly reduced. Thus, FABP4 expression was confirmed as a novel and valuable marker for determining the prognosis of GISTs, as well as an independent indicator of GIST risk (106).

#### LDLR

Cholesterol was reported as a vital structural component of cell membranes (107). Cholesterol can be *de novo* synthesized by cells or by internalizing low-density lipoprotein (LDL). LDL was found to bind to the LDLR and was internalized before entering lysosomes, where free cholesterol was released (107). LDLR-related protein 1B (LRP1B) also belongs to the LDLR family (108). LRP1B binds extracellular ligands, apoE-loaded lipoproteins, and urokinase plasminogen activator (uPAR); on that basis, it mediates the internalization of the above ligands. Impaired endocytosis of the cytoplasmic domain of LRP1B results in insufficient ligand internalization, which is correlated with poor prognosis in patients with diffuse-type gastric cancer. Cytoplasmic LRP1B immune response significantly correlated with low clinicopathological stage and good prognosis in patients with diffuse-type gastric cancer but not intestinal-type gastric cancer. Cytoplasmic LRP1B immune response is of independent prognostic value in diffuse gastric cancer, whereas it was not found to be significantly correlated with patient prognosis in intestinal-type gastric cancer (109). Chang et al. investigated cholesterol importing *via* lipoprotein/receptors (L/R route), steroidogenic enzymes, and steroid receptors in patients’ prognosis with gastric cancer. The results revealed that lipoprotein-mediated cholesterol entry and steroidogenesis are biosignatures of GC progression (50).

### *De Novo* Lipid Synthesis

#### ACSS2

The conversion of acetate to ACSSs makes acetate a vital molecule for lipid synthesis and histone acetylation. In mammalian cells, ACSS isoforms 1 and 3 are localized in mitochondria, while isoform 2 is present in the cytoplasm and nucleus (110). SREBPs regulate the expression of isoform 2. Mammalian ACSS2 was first cloned as a target of the SREBP transcription factor regulating lipid homeostasis (111). By exposing mice to a high-fat diet (HFD) or prolonged fasting, it was found that this simple metabolic enzyme could promote the proper storage or utilization of fat depending on the fed or fasted state. Thus, ACSS2 was found to play an unexpected role in controlling systemic lipid metabolism (112). Only ACSS2 down-regulation significantly inhibited acetate-mediated lipid synthesis and histone modification. In clinical practice, the deficiency of ACSS2 expression could serve as an independent prognostic factor predicting poorer prognosis in patients with gastric cancer, which was found to be especially correlated with Microsatellite Instability (MSI) (19).

#### ACLY

ACLY is a recognized vital enzyme in the *de novo* synthesis of fatty acids, and ACLY catalyzes the conversion of citrate to acetyl-CoA. It is a vital source of energy for the growth and metabolism of cancer cells. The ACLY-mediated conversion of citrate to acetyl-CoA is the basis for fatty acid synthesis (113). The protein released by HP results in the up-regulation of ACLY gene expression, promotes the synthesis of acetyl-CoA, and enhances the interaction between H. pylori (HP) and gastric epithelial cells, eventually leading to gastritis and epithelial damage (114). The study by Cheng et al. found that the overexpression of miR-133b could reduce the proliferation and invasion ability of gastric cancer cells by inhibiting the expression and activation of ACLY (114). The study by Qian et al. found that high expression of ACLY was correlated with overall survival, stage, and lymph node metastasis. ACLY can serve as a biomarker for predicting disease progression and prognosis in patients with gastric adenocarcinoma (17).

#### ACC

ACC are enzymes that catalyze the carboxylation of acetyl-CoA to form malonyl-CoA. In mammals, ACC1 and ACC2 are the two members of ACCs. ACC1 is localized in the cytoplasm and is the first rate-limiting enzyme in the *de novo* fatty acid synthesis pathway. ACC2 localizes to the mitochondrial outer membrane, produces malonyl-CoA, and regulates the activity of CPT1 involved in fatty acid β-oxidation (115). Increased activation of ACC is ordinary in human gastric cancer. pACC down-regulation is a significant prognostic factor, and pACC deficiency may play a vital role in developing GC. High pACC expression strongly correlated with better survival in all gastric cancer patients. Low/absent expression of pACC was significantly correlated with (1) advanced tumor stage and lymph node metastasis (2); poor GC cells differentiation status (3); shorter survival than those with high pACC expression, especially at an early stage. In addition, metformin inactivated ACC, which was characterized by ACC phosphorylation and significantly inhibited cell proliferation and growth (21). ACC expression in GC was significantly negatively correlated with immune signals. Specifically, the expression of ACC was significantly negatively correlated with the infiltration level of CD8+ T cells and immune cytolytic activity in GC, which suggested that inhibiting ACC could enhance antitumor immunity in gastric cancer. ACC might be a valuable biomarker to differentiate the response to immunotherapy in gastric cancer patients or a promising intervention target to enhance anti-tumor immunity and immunotherapy response in gastric cancer (20).

#### FASN

FASN refers to the major enzyme involved in the anabolic conversion of dietary carbohydrates to fatty acids. By using acetyl-CoA as a primer, malonyl-CoA as a two-carbon donor, and NADPH as an intermediate reducing agent, FASN catalyzes the synthesis of long-chain lipids fatty acids, which largely covers synthetic palmitate (80%), myristate (10%), as well as stearate (10%). FAS is highly expressed in gastric cancer, adenoma, regenerating epithelium, as well as intestinal metaplasia. FAS expression occurs at an early stage of tumorigenesis, it plays a vital role in forming precancerous lesions in gastric cancer (116). FASN is overexpressed in GC tissues, correlated with worse survival outcomes, and largely contributes to GC carcinogenesis. FASN expression was closely correlated with the immune infiltration levels of CD8+ T, CD4+ T, neutrophils, macrophages, and dendritic cells. FASN is an oncogene in GC and can serve as a potential biomarker. Existing studies have found that FASN is significantly overexpressed in GC tissues, and its high expression leads to poor survival outcomes in GC patients. Furthermore, FASN expression is significantly correlated with immune infiltration and may play a vital role in GC-related immunology (117). GC cells are highly resistant to Anoikis, and Anoikis resistance promotes the proliferation, migration, and invasion of GC cells while inhibiting GC cell apoptosis. Down-regulation of FASN has an inhibitory effect on Anoikis resistance (AR) in GC cells and is correlated with the inhibition of the p-ERK1/2/Bcl-xL signaling pathway. In brief, as revealed by the results and data of this study, FASN may be a novel target for preventing tumor metastasis, which provides a new reference for anticancer therapy (118).

#### SCD1

SCD1 refers to a novel endoplasmic reticulum-related enzyme synthesizing fatty acid synthase (FAS), which catalyzes the desaturation of saturated fatty acids (SFAs) to Δ9monounsaturated counterparts (MUFAs) (e.g., Stearic acid (18:0) and palmitic acid (16:0) yield oleic acid (18:1) and palmitoleic acid (16:1) (119)). Both SFAs and MUFAs are significant components of human cellular lipids, essential components of biological membranes, and sources of energy and signaling molecules (e.g., cholesteryl esters) (120). SCD1 enhances GC stemness *via* the Hippo/YAP pathway. SCD1 inhibition reduced the migratory/invasive ability and wound healing ability of GC cells. SCD1 overexpression is correlated with mesenchymal markers in GC tissues. After SCD1 down-regulation, GC cells show a mesenchymal-to-epithelial transition phenotype. YAP and TEAD1, the levels of essential proteins of the Hippo signaling pathway are correlated with SCD1 expression, and SCD1 inhibition leads to YAP inhibition (121). In addition, SCD1 down-regulation reduced p-YAP expression, leading to YAP disassembly in the GC nucleus, which also attenuated the activation of the Hippo pathway. Furthermore, SCD1 plays a crucial role in regulating lipid metabolism and ferroptosis in gastric cancer stem cells (GCSCs) (122). SCD1 is capable of accelerating the migration of gastric cancer cells, inhibiting cell death and growth, as well as predicting a poorer prognosis for gastric cancer patients. Thus, the potential of SCD1 as a biomarker for early diagnosis and a therapeutic target for gastric cancer has been demonstrated (23).

### Elongation of Very Long-Chain Fatty Acid Protein 5 (ELOVL5) and Fatty Acid Desaturase 1 (FADS1)

The elongation and desaturation reactions of PUFAs in cells are dependent on the expression of elongates and desaturases. Intracellular PUFAs can be elongated by the extension of the elongates of very long-chain fatty acids (ELOVLs). Desaturation reactions are catalyzed by FADS1 and FADS2 gene products (123). Lee et al. found that ELOVL5 and FADS1 were up-regulated in mesenchymal gastric cancer cells, leading to ferroptosis sensitization. In contrast, DNA methylation silences the above enzymes in intestinal-type GCs, rendering the cells resistant to ferroptosis (124).

#### SREBPs

SREBPs refer to a family of basic-helix-loop-helix leucine zipper transcription factors that regulate *de novo* synthesis of fatty acids and cholesterol and cholesterol uptake. Mammalian cells express three SREBP proteins, SREBP-1a, -1c, and -2, which are encoded by two genes, sterol regulatory element-binding transcription factor 1(SREBF1), as well as sterol regulatory element-binding transcription factor 2(SREBF2). SREBF1 encodes SREBP-1a and -1c proteins through selective transcriptional start sites. SREBP-1a protein nh2 terminal is approximately 24 amino acids longer than -1c and is highly transcriptional active. SREBP-1a regulates the synthesis of fatty acid and cholesterol as well as cholesterol uptake, while SREBP-1c mainly controls fatty acid synthesis. SREBF2 encodes the SREBP-2 protein and plays a vital role in regulating cholesterol synthesis and uptake (125). The study by Zhao et al. confirmed that SREBP-1a could bind to the GPX4 promoter region to promote the transcription of glutathione peroxidase 4(GPX4) in gastric cancer cells. Apatinib inhibited the transcription of GPX4 by regulating the srebp1a-mediated pathway and culminating in lipid peroxidation-induced ferroptosis in gastric cancer (126). Sun et al. found that SREBP-1c was activated in human gastric cancer tissues. Furthermore, the srebp-1c-dependent adipogenic pathway can be stimulated by human gastric cancer, promoting the expression of a series of fatty acid synthesis-related genes, such as SCD1 and FASN. Knockdown of the SREBP1c gene can significantly inhibit the proliferation, invasion, and migration of gastric cancer cells, thus providing novel evidence supporting SREBP-1c as a potential therapeutic biomarker target for gastric cancer. In addition, low expression of the SREBP-1c gene was correlated with better 5-year survival rates in gastric cancer patients, and most patients with stage III gastric cancer achieved higher 5-year survival rates (25).

### Lipid Storage/Lipid Droplets

#### DGAT1

Cellular lipids in excess were converted to triglycerides and cholesteryl esters in the ER, leading to the formation of lipid droplets observed in gastric cancer (32). Diglyceride acyltransferase 1/2 (DGAT1/2) can synthesize triglycerides from diglycerides and acyl-CoA (127). This enzyme is highly expressed in gastric glands, and its expression level is negatively correlated with prognosis and patient survival (27).

#### Sterol o-Acyltransferase 1 (SOAT1)/Acyl-CoA Acyltransferase 1 (ACAT1)

SOAT1, i.e., ACAT1, can convert cholesterol into cholesterol esters and store them in lipid droplets. SOAT1 regulates the expression of cholesterol metabolism genes SREBP1 and SREBP2, which induce lymph angiogenesis by increasing the expression of vascular endothelial growth factor-C (VEGF-C). SOAT1 is highly expressed in cancerous tissues and correlated with advanced tumor stage and lymph node metastasis, leading to a poor prognosis of GC (49).

### Cholesterol Synthesis

#### HMGCR

Cholesterol synthesized from mevalonate plays a crucial role in forming and maintaining cell membrane structure and function. It is also a precursor to steroid hormones, vitamin D, and bile acids (128). The mevalonate pathway itself is regulated by transcriptional and translational machinery (129). The rate-limiting step in cholesterol production is mediated by HMGCR, making it the most controlled part of the pathway (130). Li et al. found that HMGCR positively regulates the oncogenic role of the Hedgehog/Gli1 signaling pathway in gastric cancer. Overexpression of HMGCR promoted the growth and migration of gastric cancer cells, whereas the down-regulation of HMGCR expression had an inhibitory effect. In addition, HMGCR also activated the transcriptional activity of Gli1. The above results suggest that HMGCR may play a vital role in the development of gastric cancer, and targeting it in therapy will have excellent effects (43).

### Fatty Acid Oxidation

#### CPT1A and CPT1C

Carnitine palmitoyltransferase (CPT), including CPT1 and CPT2, plays a critical role in FAO. CPT1, located in the outer layer of the mitochondrial membrane, is considered a vital enzyme in FAO that converts carnitine to fatty acylcarnitine (131, 132). CPT1 consists of three isozymes (e.g., CPT1a, CPT1b, and CPT1c). CPT1c is inactive, while CPT2 is located in the mitochondrial membrane, where it promotes the β-oxidation of fatty acids (FAs) by facilitating the conversion of acetyl coenzyme A (CoA) to fatty acid coenzyme A. CPT takes on a critical significance in the oxidation of long-chain FAs (133). CPT1A protein expression is correlated with the grade, pathological stage, lymph node metastasis, as well as poor prognosis in patients with GC (68). High expression of CPT1C induced by hypoxia is closely correlated with poor prognosis (69).

### Lipolysis

#### ATGL and MGLL

ATGL initiates the lipolytic catabolism of TGs by converting TGs to DGs and FAs. The subsequent steps of lipolysis are catalyzed by hormone-sensitive lipase (HSL), which hydrolyzes DGs into MG and FAs. Lastly, mg is hydrolyzed to FAs and glycerol by monoacylglycerol lipase (MGL) (134). The study by Al-Zoughbi et al. found that low levels of ATGL mRNA were correlated with significantly reduced survival in gastric cancer patients. MGLL mRNA levels were significantly increased from adjacent normal tissues to non-high-risk groups, and gastrointestinal stromal tumors were significantly elevated and correlated with immune expression levels from non-high-risk to high-risk groups (37). MGLL overexpression strongly predicted poorer disease-free survival and overall survival. In brief, MGLL is a lipid-metabolizing enzyme correlated with the progression of gastrointestinal stromal tumors with adverse clinicopathologic factors and independent adverse prognostic effects (39).

## The Limitations of the Quality of Lipid Biomarkers

Clinically applicable biomarkers for gastric cancer are required for early detection of the disease, as well as for accurate diagnosis, prognostic stratification, and post-treatment monitoring of gastric cancer. We hope that vital enzymes of lipid metabolism can serve as biomarkers to assist clinicians when predicting the survival and prognosis of GC patients and facilitate personalized treatment. However, current evidence supporting the association of lipid biomarkers is experimental and usually retrospective, leading to significant limitations on their application. It is generally considered that an effective biomarker of malignancy should exhibit specific characteristics. To be specific, it should be detectable at high levels in cancer-affected patients and undetectable or present at low levels otherwise. Besides, it should be easily quantifiable in clinical samples and demonstrate functional correlation with disease progression to provide prognostic or diagnostic information (135, 136). However, little is known about the lipid metabolism-related pathways and the dynamic regulation of critical enzymes in the regulation of gastric carcinogenesis. As a result, the development of lipid biomarkers remains at an early stage, safety, efficacy, and adverse reactions have not been explored. The above will hinder the generalization of the biomarker-related results in clinical trials to the entire gastric cancer patient population. Furthermore, existing well-established biomarkers (e.g., Human epidermal growth factor receptor 2 (HER2) and programmed cell death-ligand 1(PD-L1)) are capable of detecting tumors early and guide specific molecule-based targeted therapy to adjust the characteristics of gastric cancer cells. The combination of lipids and conventional biomarkers will produce more accurate prediction.

## Targeting Lipid Metabolism for GC Therapy

**Figure 3:**
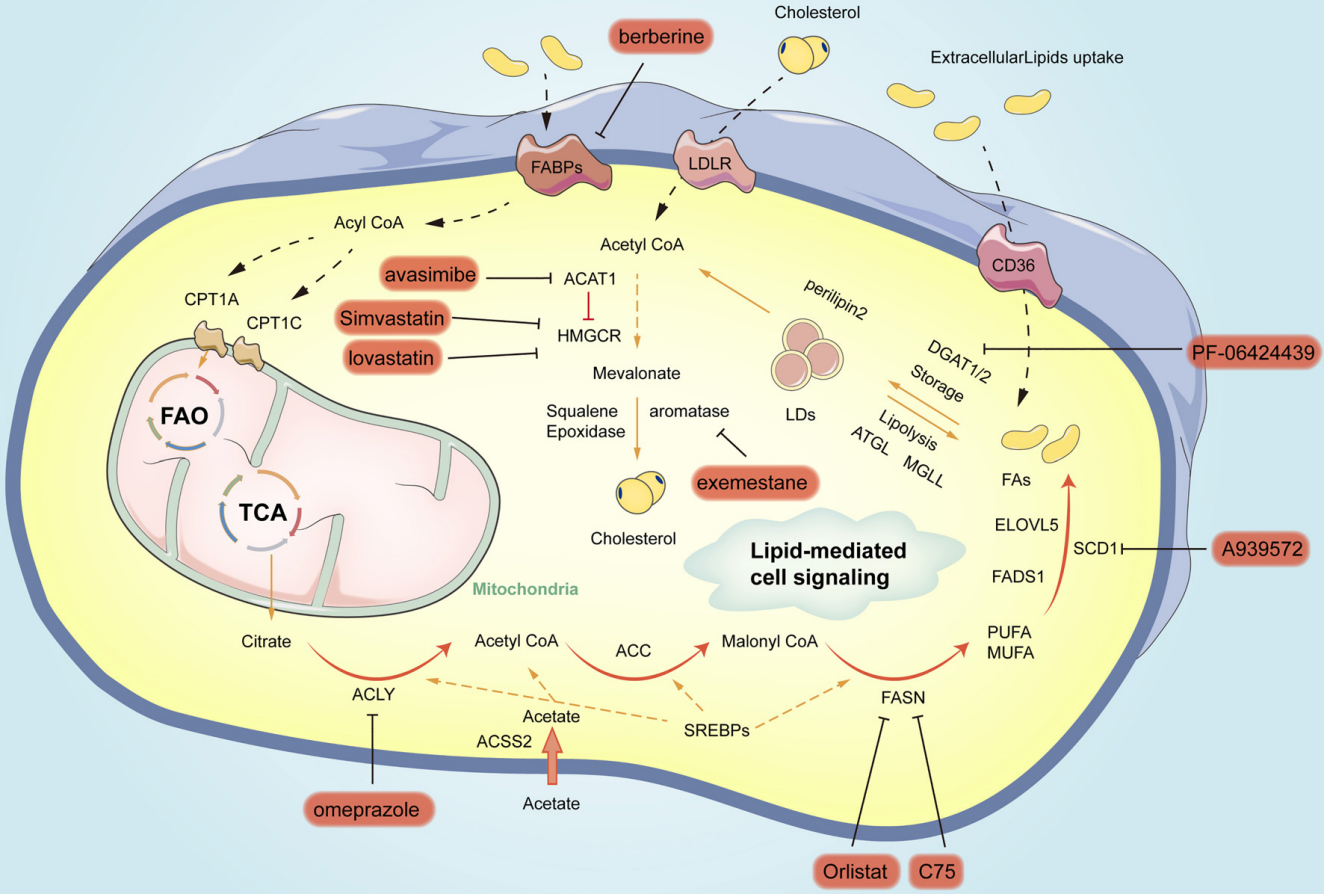
Summary of small-molecule inhibitors of lipogenic enzymes in gastric cancer. In orange circles are the names of inhibitors for different vital enzymes for blocking the related pathways.

Chemical inhibitors targeting critical enzymes of lipid metabolism in GC are currently under preclinical and clinical investigations. Representative inhibitors are listed in **Table 1** and **Figure 3**. A considerable number of natural and synthetic inhibitors of ACLY have been tested for anticancer effects in preclinical and clinical studies (146). Other drugs (e.g., proton pump inhibitors) have been employed to suppress the FASN gene in cancer, and clinical trials are being performed on one of the above drugs, omeprazole, for several cancer types. As revealed by the study of Chen et al., treating gastric cancer epithelial cells with omeprazole significantly inhibited the expression of FASN and ACLY, while inhibiting the synthesis of new lipids, which would result in lower lipid content (147). Orlistat was found as an over-the-counter anti-obesity drug reducing dietary fat absorption by covalently modifying an enzyme that inhibits gastric and pancreatic lipase (148). It was also reported as an excellent inhibitor of FAS (149). Inhibition of FAS by orlistat exposure resulted in rapid cellular damage followed by a direct but transient autophagic response. Long-term survival studies in rats and other mouse strains should be conducted to further assess orlistat’s potential to inhibit GI cancers (142). Significant efficacy of C75/imatinib combination therapy was observed on imatinib-resistant GIST cells and xenotransplantation. Exposure to C75 alone or in combination with C75/imatinib combination attenuated AKT activity (141). SCD1 and its catalytically active products are key drivers of tumor transformation and cancer progression. Accordingly, it is reasonable to consider the use of SCD1 inhibitors as possible anticancer drugs. The inhibition of SCD by a small molecule inhibitor (A939572) can impair the proliferation of gastric cancer cells (150, 151). Besides targeting *de novo* synthesis, blocking FA uptake may be another effective strategy for cancer therapy. CD36 expression is up-regulated in gastric cancer (10). Combination therapy targeting lipogenesis and lipid absorption would be a promising approach. JC61.3, an antibody targeting CD36, inhibits the uptake of FA and LDL proteins only and is potent and specific for lymph node metastases (99). JC61.3 has been tested in preclinical models of gastric cancer and found to inhibit *in vitro* cell migration and *in vivo* metastasis of gastric cancer (14). Inhibiting the ability of cancer cells to utilize FA as an energy source represents another potential therapeutic opportunity. The inhibition of lipid uptake within mitochondria to limit energy production has been an attractive therapeutic target for years. In preclinical studies, etomoxir, an irreversible inhibitor of CPT1 acting on AGS/BGC823 cells that overexpress CPT1A, has been one of the most used inhibitors of mitochondrial FAO. Inhibition of FAO with etomoxir was found to inhibit the growth, migration, and EMT process of CPT1A-overexpressing GC cells (68). Besides, the combination of oxaliplatin and perhexiline significantly inhibited the progression of gastrointestinal tumors in a patient-derived xenograft model (PDX) (137). Lipid storage has been found as a novel and vital mechanism affecting the survival and metastasis of cancer cells. Over the past few years, targeting the TAG-producing DGAT enzyme and storing it in LD has become an exciting target for obesity and type 2 diabetes research. The inhibitory effect of PF06424439 on DGAT2 has been tested in preclinical models, and PF06424439 reduced LD formation and metastasis of gastric cancer *in vitro* and vivo (28). Several inhibitors of the mevalonate acid pathway have been tested in gastric cancer, and statin cholesterol-lowering drugs have been tested in clinical trials. Simvastatin combined with Capecitable/cisplatin (CDDP) can limit the survival and growth of primary gastric cancer cells (NCT01099085) (152). Lovastatin plus docetaxel has been recognized as a promising anticancer strategy for sensitive or resistant tumors (153).Other drugs interfering with the mevalonate acid pathway (e.g., zoledronic bisphosphonate acids and aureoyl and geranyl transferase inhibitors affecting protein prenylation) have also been employed and tested in clinical trials. Avasimibe* *is a prominent anticancer drug that significantly reduces cholesteryl ester storage by inhibiting vesicule transport, integrin, and transforming growth factor beta (154). We found for the first time that avasimibe significantly inhibited the proliferation, migration, and invasion of GC cells in a dose-dependent manner. The vital enzymes of different steps of mevalonate acid have different therapeutic effects on primary and metastatic gastric cancer (distant liver metastasis). Statins and avasimibe have been considered as promising new antitumor drugs in primary gastric cancer but not in gastric cancer cells, which are highly resistant. Moreover, terbinafine (a clinically used enzyme inhibitor), which is much less enzymatic in nature than HMGCR, has been proven to be a very promising chemo-preventive natural compound, and it does inhibit monooxygenase, lower serum cholesterol. Accordingly, it can be a fascinating target for cancer prevention (45). Furthermore, exemestane (a type II aromatase inhibitor) also significantly inhibited the growth of gastric cancer cells at a pharmacologically tolerable dose *in vitro (* 50).

**Table 1:** Summary of fatty acids related targets/enzymes and their links to GC metastasis and drug resistance.

	Target/Enzymes	Description	Function	Role in GC metastasis and/or drug resistance	reference
Catabolism and uptake	CD36	transmembrane glycoprotein	absorbs extracellular lipids	fatty acid-induced CD36 expression promotes gastric cancer metastasis	(10)
	FABPs	fatty acid binding proteins	regulate fatty acid uptake, transport, and metabolism	FABP5 fosters proliferation and invasion of GC	(16)
	CPT1A	carnitine palmitoyltransferase 1A	a key enzyme of fatty acid oxidation	proliferation of GC cells metastasis	(137)
	CPT1C	carnitine palmitoyltransferase 1C		increases oxaliplatin resistance	(137)
				inhibition of FAO with etomoxir (ETX) alleviated FOLFOX regiment resistance	(138)
	ALDH3A1	aldehyde dehydrogenase 3A1	4-hydroxynonenal was converted to FAs with NADH, production by ALDH3A1, resulting in further FAO.	leads to FAO and 5-fluorouracil and cisplatin chemoresistance	(139)
*De novo* FA synthesis	ACLY	ATP-dependent citrate lyase	generate acetyl coenzyme A for *de novo* fatty acids	The expression of ACLY is associated with lymph node metastasis in GA.	(17)
	FASN	fatty acid synthase	catalyzing the conversion of acetyl-CoA and malonyl-CoA into palmitic acid	C75 suppresses increased FASN overexpression	(140)
				chemosensitivity of Imatinib	(141)
				Effective suppression of FAS and prompt destruction of membrane integrity.	(142)
	SCD1	stearyl coenzyme A desaturase enzyme 1	catalyzing the conversion of saturated fatty acids, into Δ9-monounsaturated fatty acids	Upregulates SCD1 enzyme expression enhances Cisplatin and paclitaxel chemoresistance.	(122)
				SCD1 promotes the stemness of GCSCs	(121)
	ELOVL5 and FADS1	elongation of very long-chain fatty acid protein 5	a key enzyme for *de novo* synthesis of long-chain unsaturated fatty acids	regulates GCSCs stemness by ferroptosis	(124)
		fatty acid desaturase 1	key rate-limiting enzyme of polyunsaturated fatty acids		
	SREBP1a	sterol regulatory element-binding protein 1a	regulate FAs and cholesterol synthesis	repress transcription of the human Cav1 gene to subvert the immune response	(143)
				Apatinib may induce SREBP-1a-mediated lipid peroxidation and then regulate the multi-drug-resistant GC cell.	(126)
Esterification and storage	DAGT1/2	diacylglycerol-acyltransferase 1 diacylglycerol-acyltransferase 2	involved in the formation of lipid droplets	promotes GC peritoneal metastasis	(28)
Cholesterol synthesis	PLIN2	perilipin2	associated with lipid accumulation	overexpression and knockdown of potential predictive biomarker perilipin2 in GC	(36)
	HMGCR	3-hydroxy-3-methylglutaryl-coenzyme A reductase	a key enzyme for cholesterol synthesis	suppression of HMGCR increased docetaxel chemosensitivity	(144)
	LXR	liver X receptor	regulating cancer cell proliferation and metastasis	enhances paclitaxel chemoresistance	(53)
	Exemestane		suppresses estrogen generation	increases 5-Fu chemosensitivity	(145)
	SOAT1	sterol O-acyltransferase 1	a cholesterol metabolism enzyme	promotes gastric cancer lymph node metastasis	(49)
				Avasimibe reduced cholesterol ester synthesis in GC and increased chemosensitivity.	(45)

## Further Studies Are Required to Investigate the Role of Lipid Metabolism in the Drug Treatment of Gastric Cancer

Targeting lipid metabolism is a promising therapeutic approach, but its application in the treatment of gastric cancer patients still requires extensive research and exploration.

First, the specific metabolic regulation in gastric cancer remains unclear. Accordingly, further studies on the specific mechanisms involved in the reprogramming of lipid metabolism in gastric cancer cells and their dual roles in multiple tumor-related signaling pathways are required to allow more effective identification of therapeutic targets. Further clarification of the roles of different lipid signaling molecules will provide new therapeutic opportunities for the development of anticancer drugs.

Second, numerous lipids and lipid analogs serve as critical regulators of gastric carcinogenesis. Currently, most of the information originates from studies conducted *in vitro* tumor cells or experimental animals, while there have been rare clinical studies in patients. As revealed by the results of preclinical studies, a combination therapy consisting of cytostatic, targeted therapy drugs, and lipid metabolism inhibitors would be more effective in cancer treatment (155). Whether we can use a combination of drugs to counteract the adaptation of gastric cancer cells to metabolic reprogramming or improve the effectiveness of current immunotherapy remains unclear. The synergy, safety, efficacy, side effects, and other aspects of lipid metabolism therapy and traditional anticancer drugs should be further assessed in clinical trials.

Again, relatively few molecules make it through the drug development process due to the chemical instability of enzymes, rapid metabolism in the body, and side effects in some cases. Currently, the above approaches are in their infancy, and there are few examples of the application of the above strategies. Further experimental studies are currently required to optimize the above potential lead molecules in cells and experimental animals for clinical application (155).There are no standard drugs as inhibitors of certain vital enzymes of lipid metabolism. Despite the powerful effects of natural compounds, their side effects are still concerning. To maintain the efficacy of certain biologically active alkaloids, high-frequency dosing is required, resulting in low patient compliance and even cumulative toxicity in patients. Thus, additional clinical trials and efficacy evaluations are needed to identify strategies to mitigate adverse effects when using the above natural compounds as treatments (155).

Lastly, the lipid profiles of different gastric cancer subtypes vary significantly with study designs, study subjects, methods, and analyses. Therefore, clinical studies are needed to elucidate the characteristics and patterns of lipid profiles in patients with different gastric cancer subtypes, the underlying mechanisms of lipid-related enzymes and proteins, as well as the standardized methods of targeting lipid metabolism (156).

## The Body Lipid Metabolism Influences the Inter-Cellular Lipid Metabolism in Gastric Cancer

In gastric cancer, the body’s lipid metabolism is capable of regulating the internal processes of cells and playing a certain role in cell-to-cell lipid metabolism. Interactions between cancer cells and neighboring immune cells support tumor growth and development by altering lipid metabolism (157). Yuan et al. confirmed that in the gastric cancer microenvironment, tumors could up-regulate the expression of Forkhead transcription factor 3 (Foxp3) in tumor-infiltrating Treg cells and inhibit the proliferation of autologous CD4(+) CD25(−) T cells by producing COX-2/PGE (2) (158). Lin et al. reported that gastric adenocarcinoma cells outperformed tissue-resident memory T (Trm) cells in lipid uptake and induced Trm cell death in a gastric adenocarcinoma cell-T cell co-culture system. When the expression of FABP4 and FABP5 in gastric adenocarcinoma tumor cells was down-regulated, the expression of Fabp4/5 in Trm cells was inhibited, and the uptake of lipids by Trm cells increased, thus increasing the survival rate of Trm cells *in vitro* and vivo (159). When macrophage receptor 1(Msr1) was up-regulated in tumor exposed dendritic cells (DCs), they continued to take up surrounding cholesterol and FAs, which accumulated in the cytoplasm. The results suggest that DCs have a reduced ability to cross-express and present Ag, ultimately leading to insufficient antigen-specific activation of CD8+ T cells in tumor cells (160, 161).The study by Luo et al. confirmed that lipid accumulation in tumor-associated macrophages (TAMs) could primarily arise from the increased uptake of extracellular lipids by gastric cancer cells, thus leading to the up-regulated expression of the gamma isoform of phosphoinositide 3-kinase (PI3K-γ). As a result, TAMs could be M2-like spectral polarization, which is manifested as decreased phagocytic ability and enhanced anti-tumor immunity (162).

## Lipid Metabolism Helps Maintain Drug Resistance in Gastric Cancer

Chemotherapy is the preferred treatment for patients with advanced gastric cancer. 5-Fluorouracil (5-FU) -based chemotherapy regimens, such as 5-FU combined with cisplatin or oxaliplatin, are generally considered first-line treatment options for advanced gastric cancer (163). However, drug resistance reduces the effectiveness of chemotherapy and triggers treatment failure. Based on the role of gastric cancer cells in chemotherapy resistance, tumor recurrence, and metastasis, novel strategies in cancer therapy should be urgently developed to eradicate this aggressive cell population.

Currently, changes in lipid metabolism are critical mediators of antitumor drug resistance. Reprogramming of lipid metabolism in drug-resistant cancer cells involves altered pathways of new fat production and lipid breakdown. Gastric cancer drug resistance appears to be correlated with the up-regulation of endogenous lipogenesis or lipolytic enzyme expression. This study reviews the correlation between the changes of lipid metabolism and drug resistance in gastric cancer and its significance, as well as methods to reduce drug resistance by abnormal lipid metabolism.**(** **Tables 1** **–** **5** lists the evidence from studies demonstrating the correlation between lipid metabolism and drug resistance in cancers).

**Table 2:** Summary of targeting phosphatidylcholine metabolism for drug resistance.

Target	Description	Function	Role in drug resistance	References
	crocodile choline	cell cycle arrest at the G2/M phase through attenuating the expressions of cyclins, Cyclin B1, and CDK-1.	Combining Notch1 inhibitors with crocodile choline might represent a novel approach for GC.	(164)
PLD1/2	Phospholipases D1, 2	catalyzes the hydrolysis of phosphatidylcholine to generate phosphatidic acid	Overexpression of PLD isozymes resulted in inhibition of taxotere-induced apoptotic cell death.	(165)
PLC	phospholipase C	produces diacylglycerol and phosphocholine through the hydrolysis of PC	VEGFR2 pro-proliferative effect decreased apatinib *in vivo*.	(166)
LPCAT1	lyso-PC acetyltransferases 1	regulates cholesterol metabolism	Overexpressed LPCAT1 promoted the protein ptumorigenic process.	(167)
cPLA2α	Cytosolic phospholipase A2alpha	PtdCho is hydrolyzed by phospholipase A 2, resulting in the production of lysophosphatidylcholine and arachidonic acid.	cPLA2α promotes chemotherapy efficacy.	(168)

**Table 3:** Summary of cholesterol related genes and their link to GC metastasis, EMT and/or drug resistance.

GeneSymbol	Description	Mode of action	Role in prognosis and drug resistance	References
ABCA1	ATP binding cassette transporter subfamily A member 1	cholesterol efflux pump/cholesterol transport/cholesterol homeostasis	ABCA1 is a gene signature linked to drug metabolism as a potential biomarker for predicting the prognostic risks of GA.	(169)
ABCG1	ATP binding cassette transporter subfamily G member 1	macrophage cholesterol and phospholipids transport/cholesterol homeostasis	Cisplatin resistance was found in GC with high increase of ABCG2 gene expression.	(170)
			Targeting MDR related ABC transporters promote gastric cancer chemo-resistance.	(171)
LRP1B	LDL receptor related protein 1B	regulates cholesterol accumulation in macrophages	LRP1B was mutated more frequently in GC, which is a novel predictive biomarker with a good response to immunotherapy.	(109, 172)
			Cytoplasmic LRP1B immunoreactivity was significantly related to a favorable prognosis in patients suffering from diffuse gastric cancer.	(173)
OSBPL3	oxysterol-binding protein like protein 3	involved in lipid transport, and cell signaling.	OSBPL3 is a novel driver gene stimulating the R-Ras/Akt signaling pathway and a potential therapeutic target in GC patients.	(174)
PCSK-9	proprotein convertase subtilisin/kexin Type 9	promoting LDL receptor degrading/cholesterol homeostasis	High PCSK9 expression levels in GC tissue were correlated with GC poor prognosis.	(175, 176)
PRKAG2	protein kinase AMP-activated non-catalytic subunit gamma 2	regulating *de novo* biosynthesis of FAs and cholesterol	Strong link between cancer hallmarks genes (PRKAG2).	(177)
OLR1	Oxidized Low Density Lipoprotein Receptor 1	OLR1 gene encodes the LOX-1 receptor protein	LOX-1 promoted migration and invasion of GC cells through PI3K/Akt/GSK3β pathway.	(178)
			oxLDL could activate the NF-κB signaling pathway mediated by LOX-1, and promote the lymphatic metastasis of GC.	(47)

**Table 4:** Summary of small-molecule inhibitors of lipogenic enzymes.

Target	Compound	Mechanism	Tissue distribution (and bioavailability)	Pre-clinical evidence and Clinical trials in cancer	Reference
CD36	JC61.3	anti-CD36 antibody	not applicable	inhibits GC growth and progression	(10)
FABPs	berberine	Targeting FABPs inhibitor	low oral bioavailability	Targeting FABPs triggering cell apoptosis through regulating fatty acid metabolism	(179)
ACLY	omeprazole	targeting *de novo* lipogenesis by inhibited ACLY expression	orally bioavailable	Omeprazole suppresses *de novo* lipogenesis in gastric epithelial cells.	(147)
FASN	Orlistat	irreversible binding of thioesterase domain of FASN	orally bioavailable	enhances survival from GC	(149).
	C75	C75 inhibits the PI3K/AKT/mTOR signaling pathway in lipid rafts	not applicable	c75 ameliorates imatinib-resistant GISTs	(141)
SCD1	A939572	SCD1 small molecule inhibition	not applicable	A939572 disrupts lipid homeostasis and apoptotic cell death.	(22)
DGAT	PF-06424439	DGAT2-specific inhibitor	orally bioavailable	reduces GC mesenteric metastasis and prevents LDs formation	(28)
CPT1	Perhexiline	competitive CPT1 inhibitor	orally bioavailable	the combination of oxaliplatin and perhexiline suppressed the progression of gastrointestinal cancer.	(137)
	Etomoxir	irreversible CPT1(liver and muscle isoforms) inhibitor	not applicable	Etomoxir treatment completely restricted the increase of FAO rate.	(69)
				Etomoxir attenuated FOLFOX regiment resistance *in vivo*.	(138)
HMGCR	simvastatin	HMGCR inhibitors	orally bioavailable	simvastain combined with capecitable/CDDP restricted primary gastric cancer cell viability and growth. (NCT01099085)	(152)
	lovastatin		orally bioavailable	Lovastatin plus docetaxel is effective against both sensitive and resistant tumors	(153)
Squalene Epoxidase	Terbinafine	squalene epoxidase inhibitors	orally bioavailable	In AGS cells, the resistance of Terbinafine was significant.	(45)
ACAT1	avasimibe	cholesterol esterification inhibitor	orally bioavailable	Avasimibe targeted the metabolism of cholesterol of Primary gastric tumors.	(45)
aromatase	exemestrane	type II aromatase inhibitor	orally bioavailable	exemestrane suppresses GC cell growth.	(50)

**Table 5:** Lipid key enzymes as biomarkers for gastric cancer prognosis.

Markers	Cancer Type	Name	Significance	Marker for Diagnosis or Prognosis	Reference
CD36	unknown	Duan et al.	CD36-driven lipid metabolic reprogramming and lead to tumor immune tolerance.	CD36 is associated with chronic diseases that can predispose to malignancy.	(101)
FABPs	adenocarcinomas	Hashimoto et al.	FABPs affect lipid fluxes, metabolism and signaling pathways.	High FABP3 expression is correlated with poor prognosis for patients with GISTs. High FABP4 expression is correlated with poor prognosis for patients with GISTs.	(106)
	gastric adenocarcinoma and gastric adenoma.				
					(180)
ACSS2	adenocarcinoma	HUR et al.	ACSS2 is a conserved nucleocytosolic enzyme that converts acetate to acetyl-CoA.	The loss of ACSS2 expression is associated with poor prognostic factor in GC.	(19)
ACSS3	unknown	Chang et al.	ACSS3 acts as a mitochondrial acetyl- CoA generator and also serves as a confounder of GC progression.	ACSS3 serves as a confounder of GC progression.	(181)
ACLY	adenocarcinoma	Qian et al.	ACLY is an important enzyme linking carbohydrates to lipid metabolism by generating acetyl-CoA from citrate for fatty acid and cholesterol biosynthesis.	ACLY is associated with the progression and poor prognosis of GA patients.	(17)
ACC	primary gastric cancer	Fang et al.	Acetyl-CoA carboxylases (ACC) are rate-limiting enzymes in *de novo* fatty acid synthesis, catalyzing ATP-dependent carboxylation of acetyl-CoA to form malonyl-CoA.	Low/absent expression of pACC was associated with advanced tumor stage and poor outcome for GC patients.	(21)
		He et al.		ACC is associated with immune signatures in GC, suggesting that inhibiting ACC could enhance antitumor immunity in GC.	(20)
FASN	Gastric adenocarcinoma	Ezzeddini et al.	FASN plays an important role in lipid metabolism and is associated with tumor-related signaling pathways.	FASN enzyme plays an important role in lipid metabolism and is associated with tumor-related signaling pathways.	(182)
		Duan et al.		FASN was associated with GC metastasis and survival. FASN may be a promising prognostic biomarker for GC patients.	(8)
SCD1	gastrointestinal (GI)	Wang et al.	SCD1, the enzyme that converts saturated fatty acids to Δ9-monounsaturated fatty acids.	SCD1 is associated with less-optimistic prognosis in gastric cancer patients.	(23)
SREBP-1C	Gastric adenocarcinoma	Ezzeddini et al.	SREBP1c is a transcription factor that regulates lipogenesis.	SREBP-1c protein expression may have diagnostic and prognostic values in GA patients	(182)
DGAT1	AGS	He et al.	plays an important role in the energy storage	DGAT1 in cancer tissue indicated a poor outcome in GC patients.	(27)
SOAT1	AGS	Zhu et al.	SOAT1 regulated the expression of cholesterol metabolism genes.	SOAT1 was associated with advanced tumor stage and lymph node metastasis, leading to the poor prognosis of GC.	(49)
CPT1A	unknown	Wang et al.	carnitine palmitoyltransferase 1 (CPT1) is a critical enzyme that catalyzes the carnitinylation of fatty acids for transport into mitochondria for FAO.	CPT1A protein expression was associated with poor prognosis in patients with GC.	(68)
CPT1C	unknown	Chen et al.		High expression of CPT1C was associated with poor prognosis.	(69)
ATGL	unknown	Al-Zoughbi et al.	the rate limiting enzyme in the triglyceride hydrolysis cascade.	Low levels of ATGL mRNA were associated with poor prognosis in gastric cancer patients	(37)
MGLL	gastrointestinal stromal tumors	Li et al.	MAG lipase (MGL) hydrolyzes MAG to release the glycerol backbone and release FA	MGLL was associated with poor prognosis in gastric cancer patients.	(39)

### The Effect of *De Novo* Synthesis and Decomposition of Fatty Acids on Chemotherapy Resistance of Gastric Cancer

**Figure 4:**
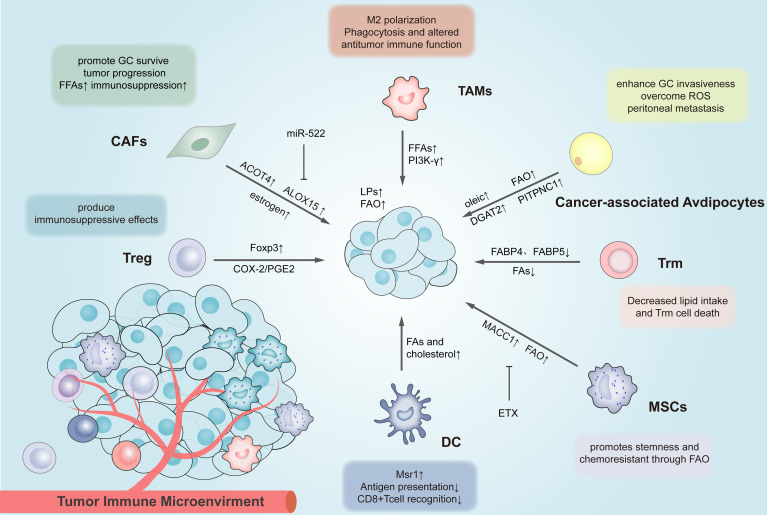
Lipid reprogramming in the tumor microenvironment affects the inter-cellular lipid metabolism and drug resistance of immune cells. Different immune cells in the TME of gastric cancer have different lipid metabolism changes, thus affecting their functions. The above metabolically reprogrammed immune cells exert different effects on GC. MSCs, Mesenchymal stem cell; CAFs, Cancer-associated fibroblasts; FFAs, free fatty acids; MACC1, Metastasis Associated in Colon Cancer 1; PITPNC1, Phosphatidylinositol transfer protein represents cytoplasmic 1; ACOT4, The acyl-CoA thioesterase 4; ALOX15, arachidonate lipoxygenase 15; Treg cells, regulatory T cells; TAMs, tumor-associated macrophages; DCs, dendritic cells; TAMs, tumor-associated macrophages; Trm cell, tissue-resident memory T cell.

Abnormal *de novo* fatty acids biosynthesis constantly supports membrane synthesis, signaling molecules, and the energy matrix of tumor cells that grow rapidly and continuously adapt to a wide variety of adverse microenvironmental conditions (e.g., limited nutrients and oxygen) (183). Lipid anabolic reorganization is capable of supporting disease recurrence and metastatic spread and changing the response to anticancer therapy, ultimately leading to drug resistance. resistance.(The above process is shown in **Figure 4**).

Cancer cells rely on increased adipogenesis to maintain their rapid proliferation, which requires high levels of anabolic lipid biogenesis for membrane synthesis. FAS is a key enzyme in the adipogenesis pathway, and its overexpression triggers cancer resistance to genotoxic drugs by increasing DNA repair. Shawn et al. reported that fatty acid synthesis was almost restricted by limiting the availability of exogenous lipids and inhibiting FAS with orlistat. Since membrane phospholipids are highly dynamic, blocking the supply of fatty acids may rapidly reduce membrane integrity, thus activating autophagy and leading to death in a short period (142). Based on the positive association of metastasis-related 1 (MACC1) in colon cancer, Duan et al. drove GC’s chemotherapy resistance to oxaliplatin by up-regulating the central enzyme formed by fat. Small interfering RNA and C75 inhibited FASN when applied experimentally, significantly reversing the down-regulation of platinum-induced chemosensitivity MACC1 overexpression. The results reveal that FASN MACC1-induced platinum resistance is a crucial link, and the inhibition of chem-resistance FASN may also serve as a therapeutic strategy (140). As the evidence demonstrating the link between the above membrane changes and drug resistance emerge, drug intervention has been focusing on critical pathways and enzymes that drive changes in the lipid profile of tumor cells. Thus, pharmacologically targeting fatty acid synthase can sensitize multiple types of cancer cells to chemotherapy *in vitro (* 184, 185) and *in vivo (* 186, 187). Zhang et al. found that IncFERO up-regulated the expression of SCD1 enzyme to adjust lipid metabolism, and iron supplementation enhanced chemotherapeutic resistance of cisplatin and paclitaxel (122). Targeting FAO as a chemosensitization strategy is also of great significance since it increases tumor cell survival by generating energy and maintaining redox balance. A recent study found that penicillin combined with oxaliplatin effectively inhibited gastric cancer progression in an *in vivo* preclinical model. In the above model, hexidine resensitized cells to oxaliplatin by inhibiting FAO and facilitating intracellular ROS accumulation (137).

Recent studies have demonstrated that cholesterol and its oxygenated derivatives could be also correlated with drug resistance in gastric cancer. Drug resistance may be due to the effect of cholesterol on cellular signaling pathways or direct effects on the expression and activity of multidrug transporters.

### Targeting Cholesterol Affecting the Activity of Drug Transporters

The acquisition of chemotherapy resistance after platinum-based therapy was regulated under the cholesterol-mediated effects on ABCG2 expression. Tuvshinjargal et al., found that patients with gastric cancer acquired cisplatin resistance, and ABCG2 gene expression was significantly up-regulated (188). Wu et al. reported that MiR-129-5p was hyper-methylated, and the overexpression led to the resistance of gastric cancer cells to the chemotherapeutic drugs, including 5-FU, vincristine (VCR), and CDDP. Through bioinformatics analysis and reporter gene analysis, three members of ABC transporters (ABC subfamily B member 1 (ABCB1), ATP-binding box C member 5 (ABCC5), and ATP-Binding box G transporter 1(ABCG1)), were identified to correlate with multidrug-resistance (MDR) and as direct targets of miR-129-5p (171). Yin et al. found that the drug metabolism-related gene ABCA1 could predict the prognosis of gastric adenocarcinoma (169). Notably, ABCB1-mediated drug outflow can be changed by drug regulation of membrane fluidity (e.g., by polyunsaturated fatty acid supplementation), suggesting that clinical lipid modifiers or dietary interventions may be promising chemo-sensitization strategies. With their relatively low proportion of polyunsaturated fat acyl groups in cells, chemo-resistant tumor cells are more likely to undergo toxic lipid peroxidation (capable of inducing apoptosis and ferroptosis) in response to oxidative stress induction chemotherapy agents.

### Targeting Cholesterol-Related Signaling Pathways

#### Cholesterol Affects Drug-Resistant Cell Signaling Pathways, Thus Enhancing Chemotherapeutic Drug Retention

In gastric cancer, simvastatin can enhance capecitabine by inhibiting markers of proliferation, invasion, angiogenesis and metastasis regulated by NF-κ B (144). Oxysterol-binding protein-like protein three (OSBPL3) is involved in lipid transport, lipid metabolism, and cell signaling transduction. OSBPL3 is a novel driver gene stimulating the R- Ras/Akt signaling pathway and a potential therapeutic target in GC patients (174).

#### Cholesterol and Its Oxygenated Derivatives Linked to GC Chemoresistance

Follet et al. confirmed that effective suppression of HMGCR enhanced the chemosensitivity of docetaxel (153). As reported by Yang et al., targeted Ar inhibitors (ARIs) could inhibit the production of estrogen and enhance the chemotherapeutic sensitivity of 5-FU (145). Avasimibe is capable of inhibiting the synthesis of cholesterol esters and lipids in GC cells and increasing the sensitivity to chemotherapy (45).

### Phosphatidylcholine and Its Metabolites Contribute to Drug Resistance in Gastric Cancer

Lipid mediators from phosphatidylcholine (PtdCho) metabolism are conducive to the treatment of drug resistance. PtdCho is hydrolyzed by Phospholipases A2 (PLA2) to produce lysophosphatidylcholine (lyso-PC) and arachidonic acid (AA). Liao et al. reported that when cytosolic phospholipase A2alpha (cPLA2α) is targeted, Ras/MEK/ERK and Akt/β-catenin signaling pathways are inhibited, ultimately enhancing the effect of chemotherapy (168).COX-2 catalyzes the conversion of AA to prostaglandin E2 (PGE2). Choi et al. reported that celecoxib could overcome the chemoresistance of 5-FU in gastric cancer by modulating the activity of COX-2 (189). The study by Qiu et al. reported that the expression level of COX-2 is significantly correlated with resistance to various chemotherapeutic drugs. (e.g., vincristine, 5-fluorouracil, oxaliplatin, mitomycin, as well as epirubicin) (190). Yu et al. found that pseudoplastic acid B circumvented MDR and increased chemotherapeutic drug sensitivity by down-regulating COX-2 expression (191). LPCAT1 converts lysophosphatidylcholine (LPC) into phosphatidylcholine (PC) in the presence of acyl-CoA in the Lands’ cycle. In gastric cancer, the expression of LPCAT1 was significantly up-regulated and facilitated the proliferation, increased the survival and enhanced the migration of cancer cells (167).

PLA2G2A hydrolyzes glycerophospholipids at the sn-2 position, releasing fatty acids and lysophospholipids (192). Chen et al. proved that artificial inhibition of PLA2G2A increased the chemosensitivity of gastric cancer cells to 5-Fu (193). In addition, PtdCho is also hydrolyzed by phosphatidylcholine-specific phospholipase C (PC-PLC) and phosphatidylcholine-specific phospholipase D(PC-PLD) to glycerol diacyl (DAG) and phosphatidic acid. Cho et al. found that the overexpression of phospholipase D (PLD) could inhibit taxotere-induced gastric cancer cell death and enhance chemotherapy resistance (165).Lin et al. demonstrated that VEGFR2 has an antiproliferative effect and chemosensitivity to Apatinib in gastric cancer cells *via* the phospholipase C (PLC)- extracellular regulated protein kinases 1/2(ERK1/2)-dependent pathway (166).DAG activates the protein kinase C (PKC) pathway. PA is the key to the activity of mTOR and promotes the proliferation and survival of cancer cells. All the above decomposition products of PtdCho are significantly correlated with chemical resistance.

### Lipid Metabolism Affects Chemoresistance by Altering the Immune Microenvironment

#### Mesenchymal Stromal Cells (MSCs)

Interstitial cells in the tumor microenvironment have been recognized as a vital factor in promoting the development of chemotherapeutic resistance. As a vital part of the tumor environment, MSCs can promote chemotherapeutic resistance and help cancer cells overcome the anticancer effects of chemotherapeutic drugs by secreting protective cytokines, even generating gene mutations and changing transcriptional expression (194, 195). To be specific, MSCs promoted self-renewal and chemotherapeutic resistance of GC cells through FAO. FAO inhibitor Etomoxir (ETX) significantly reversed stem cell characteristics and resistance to 5-FU and oxaliplatin, suggesting metabolic reprogramming of GC cells when interacting with MSCs (138). Interestingly, as revealed by an existing study, MSC co-culture activated FAO in GC cells, which led to an enhancement of chemotherapeutic resistance (196).

### Cancer-Associated Adipocyte

Adipocytes refer to the extracellular source of lipids in cancer cells (197). Co-cultured adipocytes are capable of increasing the proliferation of gastric cancer cells through lipolysis and provision of FAs. The above adipocyte-derived FAs are transported to GC cells to form lipid droplets and provide NADPH for ROS elimination during peritoneal metastasis. When DGAT2 was inhibited *in vivo*, it synergized with chemotherapeutic drugs to significantly inhibit peritoneal metastasis of gastric cancer (198). Tan et al. found that phosphatidylinositol transfer protein, cytoplasmic 1 (PITPNC1), interacts with adipocytes and GC omental metastasis to promote anoikis resistance by enhancing FAO (9).

#### CAFs

As a significant part of tumor stroma, CAFs are activated during carcinogenesis. CAFs interactions with tumor cells may contribute to the development of invasive phenotypes of cancer cells, including metastatic potential and chemotherapy resistance (199). Zhang et al. suggested that arachidonic acid lipoxygenase 15 (ALOX15), the primary mediator of lipid ROS production in GC cells, was significantly down-regulated, and the sensitivity of gastric cancer cells to cisplatin and paclitaxel decreased significantly. ACOT4, a subtype of the ACOTs family, can catalyze the hydrolysis of fatty acid acyl-CoA to CoA-SH and free fatty acids. The above enzymes have been found to play crucial roles in lipid metabolism (200). CAFs are capable of inhibiting ferroptosis by targeting ALOX15 and blocking lipid-ROS accumulation and secreting exosome Mir-522 (201). As reported by another existing study, estrogen could stimulate gastric CAFs to secrete IL-6 and activate the STAT3 signaling pathway in GC cells (202). As a result, the proliferation and invasion ability of gastric cancer cells could be enhanced, and the chemotherapy resistance of GC cells was also confirmed to be crucial (203).

### Targeting Lipid Raft Formation in Gastric Cancer Cells

We recently reported that the anti-epidermal growth factor receptor (EGFR) -targeted drug cetuximab facilitated the formation of DISC in gastric cancer cell lipid rafts, which promoted TNF-related apoptosis-inducing ligand (TRAIL)-induced apoptosis (204). It was found that β-element increased the anticancer activity of doxorubicin in multidrug-resistant gastric cancer cells by down-regulating Akt phosphorylation and P-GP expression (205). As revealed by recent studies, oxaliplatin, cisplatin, epirubicin, 5-fluorouracil and other chemotherapy drugs could increase the sensitivity of gastric cancer cells to TRAIL (206–208). Furthermore, keratin 6 regulated lipid raft formation, thereby enhancing the chemotherapy resistance to cisplatin (209).

### Lipid Metabolism Alters Chemotherapy Resistance by Affecting Autophagy in Gastric Cancer

Autophagy is a significant mechanism leading to chemotherapy resistance in gastric cancer. Autophagy could protect gastric cancer cells from the cytotoxicity of chemotherapeutic drugs and promote the formation of chemotherapy resistance (210).Lipid metabolism plays different roles in gastric cancer chemotherapy resistance by regulating autophagy. If properly applied, targeted autophagy may be a significant strategy for the prevention and treatment of chemotherapeutic resistance (211). COX-2 refers to a rate-limiting enzyme of PGE2 biosynthesis and is often overexpressed in helicobacter pylori infection, precancerous lesions and gastric cancer patients (212, 213). COX-2 promotes carcinogenesis by supporting the production of PGE2. PGE2, an active lipid compound derived from arachidonic acid, regulates different stages of the immune response such as chronic infections or cancer. Pro-inflammatory eicosanes were found to play a vital role in tumorigenesis by transducing their signals through EP1-4 and PGE2 and facilitating cell proliferation, angiogenesis, invasion and metastasis by inducing a sustained inflammatory response (212, 214, 215). Bacterial infection and PGE2 signaling are required for gastric tumorigenesis in mice; they jointly up-regulate CC-chemokine ligand 2 (CCL2), in which macrophages are recruited to gastric tumors. The expression of CCL2 is correlated with invasion, metastasis, and drug resistance of tumors. It was found that DDP-resistant gastric cancer cell lines (e.g., BGC823/DDP cells and SGC7901/DDP cells) secreted more CCL2 to maintain DDP resistance (216, 217). Moreover, CCL2 overexpression up-regulated the expression of p62 by activating the PI3K/AKT/mTOR signaling pathway. The increase in p62 expression activated the transcription of CCL2, inhibited autophagy, and formed a positive feedback loop to maintain drug resistance (218). Chi et al. initially demonstrated the methylation of MGMT promoter in gastric tissue through COX-2/PGE2 axis in COX-2 transgenic mice (219).

As reported by Lei et al., a high expression of promoter methylation of the DNA repair gene O-methylguanine DNA methyltransferase (MGMT) was significantly correlated with a low expression of autophagy-related gene 4B (ATG4B), which revealed a reasonable prognosis of gastric cancer. DDP was found to down-regulate MGMT expression in a dose- and time-dependent manner, and a low MGMT expression could induce autophagy and cisplatin resistance. Overexpression of MGMT could inhibit autophagy and reverse DDP resistance *in vivo* and *in vitro* (220). As revealed by the results, the COX-2 specific inhibitor celecoxib or the non-selective COX-2 inhibitor sulindac could synergistically inhibit GC with decitabine *in vitro* and *in vivo* (219). Indomethacin, a non-steroidal anti-inflammatory drug, was reported as an adjunct to anticancer drugs with satisfactory efficacy (221, 222). Vallecillo-Hernandez et al. suggested that indomethacin induces accumulation of p62 and BRCA1 (NBR1) neighbors in AGS cells, impairs lysosomal function, inhibits autophagic degradation, and increases oxa-induced cell death (211). It was reported that oral administration of LPA lipids in Chinese herbal medicines could increase the production of prostaglandin E2, thus leading to a high expression of LPA2 in gastric cancer cells. The above could prevent indomethacin-induced cell death and stimulate the proliferation of gastric cancer cells (223).

### Lipid Metabolism Contributes to Chemoresistance by Causing Oxidative Stress in GC

Lipid droplets were found to decrease ROS toxicity, thus increasing cancer cell survival (224). In numerous types of cancer, LDs facilitated tumor growth, while enhancing metastasis, chemotherapy resistance, as well as disease recurrence (225–227). Lipid droplets can act as additional lipid sources for fatty acid oxidation under nutritional stress or as “precipitates” for isolating hydrophobic drugs, leading directly to chemotherapy resistance (228). For instance, tumor cell lines with high LDs content have increased chemotherapy resistance to 5-FU and oxaliplatin. Stimulated by lysophosphatidylcholine acyltransferase 2 (LPCAT2), the above cells further accumulated LPCAT2 during the chemotherapy response, and the increased expression of LPCAT2 prevented the endoplasmic reticulum stress induced by chemotherapy, further highlighting the influence of LDs on the cell chemotherapy response (226, 229). Enjoji et al. reported a significant accumulation of lipid droplets in gastric cancer cells, primarily in the formation of new lipid and lipid storage (26). As reported by Choi et al., ROS levels in gastric cancer cells increased dose-dependently after 5-FU treatment, while ROS levels in GC were maintained at a relatively low level. GC was found with increased OXPHOS and paradoxically maintained a low ROS level through FAO-mediated NADPH regeneration (230). On that basis, the sensitivity to chemotherapeutic drugs (5-FU) decreased. N-3 docosahexaenoic acid (DHA) was found to induce ROS-dependent apoptosis in cisplatin-resistant gastric cancer (231).

### The Degree of Lipid Saturation Affects the Physicochemical Properties of Cell Membranes

Increased levels of saturated fatty acids make cell membranes more resistant to ROS-dependent peroxidation and cell death attributed to ferroptosis. For instance, orlistat was found to reduce the absorption of exogenous dietary fat by inhibiting gastric and pancreatic lipase, and it was also indicated as an excellent FAS inhibitor (142). Chen et al. found that LDLR-reduced LPC abundance perturbed the phospholipid homeostasis of the Lands cycle in LDs, thus reducing intracellular platinum transport during DNA adduct formation and then increasing cisplatin sensitivity in gastric cancer (232).

## Conclusions

Abnormal lipid metabolism represents a crucial component of tumor metabolic adaptation, which is conducive to enhancing treatment resistance and facilitating metastasis. In this study, the specific process of lipid metabolism reprogramming in gastric cancer is described. Moreover, the roles of lipid metabolism in gastric cancer treatment, novel biomarkers, and chemotherapy resistance of gastric cancer cells are summarized. Numerous drugs targeting lipid metabolism pathways have been developed for cancer treatment. However, the role of these drugs in gastric cancer remains unclear. Accordingly, further research is needed to gain a comprehensive and in-depth understanding of the lipid metabolism of GC.

## Author Contributions 

M-YC, D-XZ, and JW contributed to the study conception and design. Material preparation, data collection and analysis were performed by M-YC and XY. The first draft of the manuscript was written by M-YC and all authors commented on existing versions of the manuscript. All authors read and approved the final manuscript.

## Funding

This study was supported by grants from National Natural Science Foundation of China (nos. 81770212, to D-XZ) and Changzhou City Health Commission major scientific and technological projects (nos. ZD201901, to JW).

## Conflict of Interest

The authors declare that the research was conducted in the absence of any commercial or financial relationships that could be construed as a potential conflict of interest.

## Publisher’s Note

All claims expressed in this article are solely those of the authors and do not necessarily represent those of their affiliated organizations, or those of the publisher, the editors and the reviewers. Any product that may be evaluated in this article, or claim that may be made by its manufacturer, is not guaranteed or endorsed by the publisher.

## Glossary

**Table:**

GC	gastric cancer
FABPs	fatty acid-binding proteins
AKT	the protein kinase B
ACLY	ATP-citrate lyase
FABP5	fatty acid-binding protein 5
ACSS2	acetyl-CoA synthetase 2
ACC	acetyl-CoA carboxylase
FASN	fatty acid synthase
pACC	phosphorylated form of ACC
MUFAs	polyunsaturated fatty acids
SCDs	stearoyl-CoA desaturases
TAGs	triacylglycerols
SREBP1	sterol regulatory element-binding protein 1
SREBP-1c	sterol regulatory element-binding protein 1 c
ELOVL6	fatty acid elongase 6
PA	phosphatidic acid
FAs	fatty acids
FFAs	free fatty acids
FAS	fatty acid synthase
DGAT2	diacylglycerol O-acyltransferases 2
TGs	triglycerides
CEs	cholesteryl esters
DGAT1/2	diacylglycerol O-acyltransferase 1/2
SOAT1	sterol O-acyltransferase 1
ACAT1	acetyl-CoA acyltransferase 1
ROS	reactive oxygen species
PLINs	perilipins
ARDP	adipose differentiation-related protein
ATGL	adipose triglyceride lipase
DAG	diacylglycerol
HSL	hormone-sensitive lipase
MAG	monoacylglycerol
MAGL	monoacylglycerol lipase
MGLL	monoglyceride lipase
GISTs	gastrointestinal stromal tumors
LSR	lipolysis-stimulated lipoprotein receptor
JAK/STAT	Janus kinase/signal transduction and activator of transcription
PI-3K	phosphatidylinositol-3-kinase
VLDL	very low-density lipoprotein
HMG-CoA	3-hydroxy-3-methylglutaryl coenzyme A
HMGCR	3-hydroxy-3-methyl-glutaryl-CoA reductase
FPP	farnesyl diphosphate
SQLE	squalene epoxidase
LDLR	low-density lipoprotein receptor
ox-LDL	oxidized low-density lipoprotein
VEGF-C	vascular endothelial growth factor-C
LOX-1	lectin-like oxidized low-density lipoprotein receptor-1
NF-Kb	the nuclear factor Kb
CD36	cluster of differentiation 36
ABC	ATP-binding cassette
ABCA1	ATP-binding cassette transporter A1
ABCGs	ATP-Binding Cassette G transporters
ACATs/SOATs	acyl-CoA: cholesterol acyltransferases
SREBP2	sterol regulatory element-binding protein 2
LXRα	the oxysterol receptor liver X receptor α
LXRβ	the oxysterol receptor liver X receptor β
LXRs	liver X receptors
ATF4	activating transcription factor 4
FAO	fatty acids oxidation
ACSL4	acyl-CoA synthetase long-chain family member 4
ALOX-15	arachidonate 15-lipoxygenase
FPR1	the formylpeptide receptor-1
GPCR	G-protein coupled receptor
RRRs	pattern recognition receptors
15-LOX-1	15-lipoxygenase-1
PPAR-g	peroxisome proliferator-activated receptor-g
cox-2	cyclooxygenase-2
NADH	reduced nicotinamide adenine dinucleotide
FADH2	reduced flavin adenine dinucleotide 2
ETC	electron transport chain
HIF-1α	hypoxia-inducible factor-1α
PPARγ	peroxisome proliferator-activated receptor γ
CPT1	carnitine acyltransferase 1
EMT	epithelial-mesenchymal transition
CPT1A	carnitine acyltransferase 1 A
CPT1C	carnitine acyltransferase 1 C
ACOT	the acyl-CoA thioesterase
ACOT4	the acyl-CoA thioesterase 4
PI3K/AKT/mTORC1	phosphoinositide 3-kinase (PI3K)-protein kinase B (AKT)-mechanistic target of rapamycin (mTOR) signaling
LIPIN1	lipin1
YAP/TAZ	yes-associated protein/transcriptional coactivator with PDZ-binding motif
LATS2	large tumor suppressor 2
GGPP	geranylgeranyl pyrophosphate
PTCH	protein patched homolog
SMO	smoothened
CRD	cysteine-rich domain
HH	hedgehog
SHH	sonic Hedgehog
CAFs	cancer-associated fibroblasts
FABPs	fatty acid-binding proteins
SFAs	saturated fatty acids
LPCAT1	lysophosphatidylcholine acyltransferase 1
PDX	patient-derived xenograft models
CDDP	cisplatin
5-FU	5-Fluorouracil
TGF-β	transforming growth factor β
MACC1	metastasis-related in colon cancer 1
VCR	vincristine
ABCB1	ABC subfamily B member 1
ABCC5	ATP-binding cassette subfamily C member 5
ABCG1	ATP-Binding Cassette G transporter 1
MDR	multidrug-resistance
PtdCho	phosphatidylcholine
lyso-PC	lysophosphatidylcholine
AA	arachidonic acid
PGE2	prostaglandin E2
PLA2	phospholipases A2
cPLA2α	ctosolic phospholipase A2α
LPC	lysophosphatidylcholine
PC	phosphatidylcholine
PC-PLC	phosphatidylcholine-specific phospholipase C
PC-PLD	phosphatidylcholine-specific phospholipase D
PLD	phospholipase D
VEGFR2	vascular endothelial growth factor receptor 2
ERK1/2	extracellular regulated protein kinases 1/2
PLC	phospholipase C
PKC	protein kinase C
MSCs	mesenchymal stromal cells
ETX	etomoxir
PITPNC1	phosphatidylinositol transfer protein
cytoplasmic 1	
EGFR	epidermal growth factor receptor
TRAIL	TNF-related apoptosis-inducing ligand
CCL2	cc-chemokine ligand 2
MGMT	Promoter methylation of the DNA repair gene O-methylguanine DNA methyltransferase
ATG4B	autophagy-related gene 4B
NSAIDs	nonsteroidal anti-inflammatory drugs
LPA	lysophosphatidic acid
DHA	docosahexaenoic acid
LPCAT2	lysophosphatidylcholine acyltransferase 2
LRP1B	LDLR-related protein 1B
uPAR	urokinase plasminogen activator
L/R	lipoprotein/receptors
HFD	high-fat diet
MSI	microsatellite Instability
HP	H. pylori
ICA	immune cytolytic activity
AR	anoikis resistance
GCSCs	gastric cancer stem cells
ELOVL5	elongation of very long-chain fatty acid protein 5
FADS1	fatty acid desaturase 1
ELOVLs	elongases of very long-chain fatty acids
SREBF1	sterol regulatory element-binding transcription factor 1
SREBF2	sterol regulatory element-binding transcription factor 2
GPX4	glutathione peroxidase 4
HER2	human epidermal growth factor receptor 2
PD-L1	programmed cell death-ligand 1
Foxp3	forkhead transcription factor 3
Trm cell	tissue-resident memory T cell
DCs	dendritic cells
TAMs	tumor-associated macrophages
PI3K-γ	gamma isoform of phosphoinositide 3-kinase
